# Corticomotor Control of Lumbar Erector Spinae in Postural and Voluntary Tasks: The Influence of Transcranial Magnetic Stimulation Current Direction

**DOI:** 10.1523/ENEURO.0454-22.2023

**Published:** 2024-02-08

**Authors:** Mikaël Desmons, Amira Cherif, Antoine Rohel, Fábio Carlos Lucas de Oliveira, Catherine Mercier, Hugo Massé-Alarie

**Affiliations:** ^1^Center for Interdisciplinary Research in Rehabilitation and Social Integration (Cirris), CIUSSS de la Capitale-Nationale, Quebec City, Quebec G1M 2S8, Canada; ^2^Rehabilitation Department, University Laval, Quebec City, Quebec G1V 0A6, Canada, G1V 0A6

**Keywords:** anticipatory postural adjustment, low back muscle, primary motor cortex, stretch reflex, transcranial magnetic stimulation, voluntary control

## Abstract

Lumbar erector spinae (LES) contribute to spine postural and voluntary control. Transcranial magnetic stimulation (TMS) preferentially depolarizes different neural circuits depending on the direction of electrical currents evoked in the brain. Posteroanterior current (PA-TMS) and anteroposterior (AP-TMS) current would, respectively, depolarize neurons in the primary motor cortex (M1) and the premotor cortex. These regions may contribute differently to LES control. This study examined whether responses evoked by PA- and AP-TMS are different during the preparation and execution of LES voluntary and postural tasks. Participants performed a reaction time task. A Warning signal indicated to prepare to flex shoulders (postural; *n* = 15) or to tilt the pelvis (voluntary; *n* = 13) at the Go signal. Single- and paired-pulse TMS (short-interval intracortical inhibition—SICI) were applied using PA- and AP-TMS before the Warning signal (baseline), between the Warning and Go signals (preparation), or 30 ms before the LES onset (execution). Changes from baseline during preparation and execution were calculated in AP/PA-TMS. In the postural task, MEP amplitude was higher during the execution than that during preparation independently of the current direction (*p* = 0.0002). In the voluntary task, AP-MEP amplitude was higher during execution than that during preparation (*p* = 0.016). More PA inhibition (SICI) was observed in execution than that in preparation (*p* = 0.028). Different neural circuits are preferentially involved in the two motor tasks assessed, as suggested by different patterns of change in execution of the voluntary task (AP-TMS, increase; PA-TMS, no change). Considering that PA-TMS preferentially *depolarize neurons in M1*, it questions their importance in LES voluntary control.

## Significance Statement

Back muscles fulfill different roles as postural and voluntary control involving different neuronal circuits. Manipulating the electrical current direction induced by transcranial magnetic stimulation may allow the examination of different neural circuit contributions to postural and voluntary control of back muscles. In the execution of a postural task, corticospinal excitability was higher for both current directions than that for preparation. In the voluntary task, the corticospinal excitability was higher during execution than that during preparation using anteroposterior current only. Neural circuit contribution to back muscles control may depend on their role in the task performed. Our results suggest a minimal involvement of motor cortex neurons (minimally those interacting with posteroanterior current) in voluntary control of back muscles.

## Introduction

Neural control of trunk muscles is unique (Desmons et al., 2023) and necessitates proper testing. Indeed, it is critical to adapt our interventions to the different health conditions that impact its control [e.g., low back pain (Tsao et al., 2011a,b; Massé-Alarie et al., 2016a; Schabrun et al., 2017)]. Nonetheless, there are only a limited number of studies that specifically tested neural control of trunk muscles (Desmons et al., 2023), showing it is organized differently from that of hand muscles (Brinkman and Kuypers, 1973; Strutton et al., 2004). For example, stimulation of the primary motor cortex (M1) elicits a higher prevalence of ipsilateral motor-evoked potentials for trunk muscles compared with hand muscles in nonhuman primates and humans (Brinkman and Kuypers, 1973; Strutton et al., 2004). In addition, the M1 representation of trunk muscles is smaller than that of hand muscles (Montgomery et al., 2013; Boendermaker et al., 2014). Moreover, the roles of trunk and hand muscles differ: trunk muscles contribute to both postural and voluntary control (Massion, 1992) of the spine in contrast to the predominant role in fine motor control of hand muscles. For example, lumbar *erector spinae* (LES) is the *prime mover* of the spine in extension (voluntary; Gilchrist, 2003) and also plays a postural role [e.g., through anticipatory postural adjustment (Aruin and Latash, 1995)]. Postural and movement control are suggested to be separate processes. For example, the postural adjustment precedes the movement onset when the movement is self-paced, whereas it occurs mostly simultaneously with the movement onset in a reaction time (RT) task (Massion, 1992). Nonetheless, transcranial magnetic stimulation (TMS) studies of human trunk muscles also suggest some similarities with the control of hand muscles. For example, when applied over M1, TMS led to selective activation of the erector spinae muscle M1 representation with short latencies (≍15–20 ms), suggesting a monosynaptic corticospinal projection (Ferbert et al., 1992; O’Connell et al., 2007; Chiou et al., 2018a; Desmons et al., 2023) and to the observation of intracortical inhibition and facilitation mechanisms (Massé-Alarie et al., 2016b; Chiou et al., 2018a). Altogether, evidence suggests that neural control of back muscles is unique with both differences and similarities when compared with control of hand muscles and then requires specific testing in humans. Understanding of the neural control of back muscles may be improved by manipulating the TMS current direction.

The manipulation of TMS coil positioning elicits a variation in the direction of electrical current flowing in the brain; different current directions may interact with different neural circuits. Commonly, a posteroanterior (PA-TMS) current direction is used and known to depolarize neural structures of the targeted cortical area (i.e., M1 for most studies; Siebner et al., 2022). In active hand muscles, single motor unit recordings showed successive descending volleys termed indirect-waves (I-wave) induced by single-pulse TMS; the I_1_-wave (i.e., early) is produced preferentially by PA-TMS, while the I_3_-wave (i.e., later) is elicited preferentially by anteroposterior (AP)-TMS (Day et al., 1989; Sakai et al., 1997). Moreover, considering AP-TMS I-wave activity is less synchronized (Di Lazzaro et al., 2018) and has peak latencies later compared with PA-TMS, it was suggested to be caused by different sources of inputs to corticospinal cells. Therefore, PA- and AP-TMS may interact with different neural populations (Di Lazzaro et al., 2018). Recent studies suggest that an anteroposterior electrical current (AP-TMS) interacts with neural circuits of the premotor cortex/supplementary motor area (SMA) projecting to M1 (Volz et al., 2015; Aberra et al., 2020; Siebner, 2020). For example, a multimodal study using TMS and fMRI reported that AP-TMS latency was correlated with functional connectivity of left M1 with ipsilateral premotor cortex and bilateral SMA (Volz et al., 2015). In contrast, authors hypothesized that PA-TMS interacts with fibers within M1 (Siebner et al., 2022), whereas AP-TMS would interact with a range of synaptic inputs from frontal motor areas (including premotor and SMA) onto M1 (Volz et al., 2015). Furthermore, a modeling study supports that reversing the current direction from PA- to AP-TMS can spatially shift the TMS site of activation from the precentral gyrus toward (M1) more anterior area (e.g., premotor; Aberra et al., 2020). Other TMS variables assessed using single- or paired-pulse TMS of hand muscles also differ between current directions. For single-pulse TMS, longer motor-evoked potential (MEP) latencies (Sakai et al., 1997; Di Lazzaro et al., 2001) and higher motor thresholds (Orth and Rothwell, 2004; Hamada et al., 2013) compared with PA-TMS were observed. For paired-pulse TMS, AP-TMS elicits more profound inhibition with short-interval intracortical inhibition protocol (SICI—representing GABAergic cortical inhibitory interneurons; Cirillo and Byblow, 2016; Sale et al., 2016). Similar differences were observed between current directions while targeting the LES M1 representation (Desmons et al., 2021) suggesting the recruitment of two neural circuits [allegedly M1 and premotor/SMA (Di Lazzaro and Rothwell, 2014; Siebner, 2020)] that may contribute differently to LES control.

Most TMS studies targeting back muscles tested corticomotor control during the maintenance of a static motor task (e.g., maintenance of isometric trunk extension in sitting; Ferbert et al., 1992; Strutton et al., 2005; Desmons et al., 2021)—which does not necessarily test circuits involved in dynamic motor tasks (i.e., involving a movement of the trunk, e.g., concentric trunk extension)—and used PA-TMS. Recent TMS studies reported an increase in the excitability of projections to back muscles during a RT task consisting of an upper limb movement eliciting anticipatory postural adjustment of back muscles (Chiou et al., 2018a; Massé-Alarie et al., 2018; Rowland et al., 2021). The increased corticospinal excitability was accompanied by a reduction of the inhibition (induced by the PA-SICI protocol) for the agonist muscle prior to the muscle activation (without change in spinal excitability measured using cervicomedullary-evoked potential). This change in the balance between cortical inhibition and facilitation is believed to reflect the contribution of cortical neurons in the execution of voluntary movements (Reynolds and Ashby, 1999). In the case of low back muscles, an increase in corticospinal excitability prior to the anticipatory postural adjustment of back muscles seems to predominantly originate from M1 (Chiou et al., 2018a). Nevertheless, other cerebral areas such as the premotor areas/SMA have been suggested to contribute to control of anticipatory postural adjustments (Massion, 1992; Volz et al., 2015). For example, repetitive TMS over SMA delayed the timing of apparition of APAs in healthy participants (Jacobs et al., 2009). Thus, AP-TMS could be used to test the potential contribution of premotor areas/SMA in postural control of back muscles. Furthermore, most studies have used the PA current during the execution phase (Ziemann et al., 1997; Duque et al., 2014; Massé-Alarie et al., 2018; Chiou et al., 2018a; Rowland et al., 2021). It is possible that MEP elicited by AP current is modulated differently during the preparation phase because of the role of premotor in motor preparation (Takakusaki, 2017). Finally, while a few studies have examined the corticomotor control of back muscles in voluntary tasks (Nowicky et al., 2001; Chiou et al., 2016), these have been limited to static tasks (e.g., isometric back extension). An exhaustive examination of neural circuits contributing to the voluntary control of back muscles in a dynamic task using different TMS current directions has been overlooked even though differences with postural control have been suggested.

The study’s main objective was to compare the effect of different current directions of TMS (PA- vs AP-TMS) on the changes in corticospinal (using single-pulse TMS) and spinal [using a muscle tap eliciting a stretch reflex (SR)] excitability and in SICI (using paired-pulse TMS) involved in the neural control of LES during the preparation and execution of a voluntary and a postural motor task. We hypothesized (1) a greater change of corticospinal and intracortical inhibition with AP-TMS compared with PA-TMS during the postural task due to the involvement of premotor regions/SMA in anticipatory postural adjustment (Massion, 1992) and (2) a greater change of corticospinal excitability and intracortical inhibition with PA-TMS compared with AP-TMS during a voluntary task due to the predominant role of the M1 in the execution of voluntary movement (Lemon, 2008). No change in spinal excitability is expected as reported in similar dynamic tasks (Petersen et al., 2009; Chiou et al., 2018a).

## Materials and Methods

### Ethical approval

The study was approved by the Ethics Committee in accordance with the latest Declaration of Helsinki, and all participants provided their written informed consent prior to participation.

### Participants

The participants were recruited through the university mailing list and consented to participate in one or two experiments. Participants were included if they had no contraindication for TMS (e.g., any history of epilepsy, pregnancy, metal in head or jaw, medication reducing seizure threshold; Rossi et al., 2011, 2021) or if they had any major neurological, respiratory, orthopedic, circulatory disorders, or low back pain since it may influence corticomotor control of back muscles (Tsao et al., 2011a,b; Massé-Alarie et al., 2016a). In the first experiment, 15 participants (of either sex) performed an upper limb movement that elicits postural activation of back muscles (postural task). In the second experiment, 15 participants (of either sex) performed a pelvic tilt for which the back muscles are *prime movers* of the lumbar spine (voluntary task). Nine participants participated in both experiments although data from only 6 participants were analysed (see demographic characteristics in Results and in Table 1).

**Table 1.** Participant characteristics [mean (SD) or occurrence]

	Experiment 1	Experiment 2	Subset	Subset
	Postural task, *n* = 15	Voluntary task, *n* = 13	Postural task, *n* = 6	Voluntary task, *n* = 6
Gender (F, M)	(10, 5)	(7, 6)	(4, 2)	
Right-handed (*n*)	14	11	5	
Age (years)	30.27 (5.23)	30.15 (5.29)	32.33 (5.99)	
Height (cm)	170.07 (9.95)	174.08 (9.3)	174.67 (9.05)	
Weight (kg)	68.91 (11.14)	68.32 (11.65)	68.87 (11.84)	
PA-AMT (% MSO)	57.15 (9.92)	56.08 (10.95)	60.83 (12.38)	60.17 (10.36)
AP-AMT (% MSO)	63.69 (10.0)	61.85 (11.31)	67.50 (10.33)	65.50 (10.77)
Reaction time (ms)	150.14 (23.03)	156.42 (28.77)	157.50 (10.78)	143.17 (28.32)

AMT, active motor threshold; AP, anteroposterior; F, female; M, male; MSO, maximal stimulator output; PA, posteroanterior; reaction time, time between the Go signal and the onset on the lumbar erector spinae.

### Experimental study design

**Figure 1.**
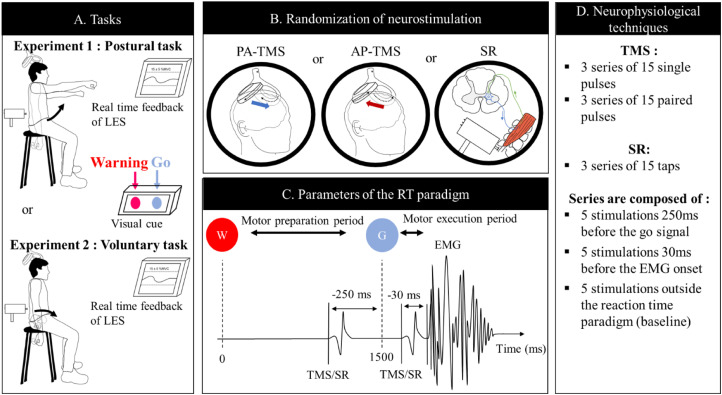
Schematic illustration of (***A***) the set-up of the two experiments, (***B***) the three types of stimulations (AP-TMS, PA-TMS, and stretch reflex), (***C***) the different parameters used during the experiments, and (***D***) the content of each series of stimulation. Note that the types of stimulations (***B***) and the content of series of stimulation (***D***) were randomized for each participant at each experiment. Panel ***A*** illustrates the performance of the postural task (Experiment 1) and of the voluntary task (Experiment 2). For the postural task (Experiment 1), participants had to perform a bilateral shoulder flexion that elicits anticipatory postural adjustment of low back muscles. For the voluntary task (Experiment 2), participants had to perform an extension of the lumbar spine by tilting the pelvis anteriorly. Participants were waiting for a Warning signal (W—orange light) followed by a Go signal (G—blue light) 1,500 ms later while participant maintained ≍15% of the maximal voluntary contraction of the back muscles. Panel ***C*** depicts the timeline of the RT paradigm. Stimulations were triggered in the motor preparation period 250 ms prior to the Go signal or in the motor execution period 30 ms prior to the EMG onset of the back muscles using a double-cone coil for TMS or a muscle tap using an electromagnetic hammer. Stimulations were randomly triggered at intervals ranging from 7 to 10 s. Panel ***D*** depicts the protocol of stimulations used: a single-pulse protocol with stimulation at 120% of AMT and a SICI paired-pulse protocol using a conditioning stimulus at 80% AMT, a test stimulus at 120% AMT, and an ISI of 3 ms. AMT, active motor threshold; AP, anteroposterior current in the brain; CT, conditioning stimulus; EMG, electromyography; ISI, interval inter stimulus; LES, lumbar erector spinae; MVC, maximal voluntary contraction; PA, posteroanterior current in the brain; RT, reaction time; SICI, short-interval intracortical inhibition; SR, stretch reflex; TMS, transcranial magnetic stimulation; TS, test stimulus.

For both experiments, a RT paradigm consisting of a Warning signal followed by a Go signal was used. Participants performed a postural (Experiment 1) or a voluntary task (Experiment 2—Fig. 1*A*) for which LES is critically involved.

#### Experiment 1: bilateral shoulder flexion (postural task)

Participants performed a rapid bilateral shoulder flexion in sitting (Fig. 1*A*). At the Go signal, participants were instructed to move their arms as fast as possible up to ≍90° of shoulder flexion. During bilateral shoulder flexion, anticipatory postural adjustment of the LES occurs in advance or time-locked to the activation of the movement agonists (anterior deltoids) to counteract the reactive force produced from the arm acceleration and the anterior movement of the center of mass and maintain sitting balance (Aruin and Latash, 1995; Massé-Alarie et al., 2018). LES is a *prime controller* of the body posture in this task (Park et al., 2014; Massé-Alarie et al., 2018)

#### Experiment 2: anterior pelvic tilt (voluntary task)

Participants performed a rapid anterior pelvic tilt in sitting accompanied by an extension of the lumbar spine (Fig. 1*A*). They were instructed to tilt their pelvis forward as fast as possible at the Go signal. During this task, LES acts as a *prime mover* of the lumbar spine extension (agonist muscles; O’Sullivan et al., 2006; Claus et al., 2009).

##### Study design for both experiments

Figure 1 illustrates the experimental design. Participants seated comfortably on a height-adjustable chair, arms along the body, and knees at ≍90° and maintained a slight LES contraction [15 ± 5% maximal voluntary contraction (MVC); Fig. 1*A*]. Muscle pre-activation was maintained at 15% MVC throughout the experiment (i.e., hotspot, motor threshold, and all experimental conditions). A computer monitor positioned in front of the participants displayed the real-time EMG feedback (window duration, 0.5 s) that helped to stabilize the subject's attention. At each trial, a diode emitting a red light served as a Warning signal, followed 1,500 ms later (fixed interval) by a blue light used as a Go signal (Massé-Alarie et al., 2018; Neige et al., 2018). The time between the Warning and the Go signals represents the preparation period, while the time after the Go signal represents the motor execution period (Duque et al., 2010, 2012). Participants were instructed to perform one of the motor tasks (described in the previous section) as fast as possible at the Go signal. Since the task was challenging to perform for some participants, a period of training was realized at the beginning of each experiment until the participants successfully performed the task five times in a row.

TMS outcomes [MEP amplitudes, SICI (conditioned MEP)] were measured using two current directions (PA- and AP-TMS), and spinal excitability was measured using a muscle tap eliciting a SR of the LES (Fig. 1*B*). Current directions were tested in separate blocks using independent hotspot and motor threshold (Fig. 1*B*). TMS or muscle taps were triggered in three different conditions: (1) during the “preparation period” (−250 ms prior the Go signal), (2) during the “execution period” (−30 ms prior to the LES EMG onset elicited by the motor tasks—procedure explained later in this section), and (3) while participants were waiting for the Warning signal (i.e., outside the RT paradigm; Fig. 1*C*). The latter was used as the baseline to normalize the MEPs recorded in the preparation and execution periods. This baseline provided a control for potential shift in excitability due to attention and preparedness to move. TMS/SR were tested during the motor preparation and execution periods since they have been shown to impact differently on MEP amplitude and represent different mechanisms (Massé-Alarie et al., 2018). The timing of stimulation during the execution period was set individually for each participant 30 ms prior to the LES EMG onset. This timing was selected since MEP amplitude was increased at 40 ms and 30 ms prior to back muscles onset (Chiou et al., 2018a) and from 75 ms prior to soleus onset (Petersen et al., 2009) elicited by different postural tasks. Also, considering that the MEP latency ranges between 15 and 20 ms (Ferbert et al., 1992; O’Connell et al., 2007; Desmons et al., 2021) and to avoid MEPs falling within EMG burst, using a timing closer to the EMG onset would have resulted in data loss. Prior the beginning of the experimental task, the LES EMG onset was measured in combination with a TMS click to replicate the experimental context in which TMS was triggered during the RT paradigm. The experimenter holds the coil at ≍30 cm of the head of the participant to avoid neuron depolarization, and the TMS intensity was set at 60% maximal stimulator output (MSO). RT was measured using one block of 15 TMS (5 in the preparation period, 5 in the execution period, 5 baseline) mimicking the experimental design and involving 10 movements. The LES EMG traces elicited by the 10 movement trials (5 with stimulations 250 ms before and 5 with stimulations 100 ms after the Go signal with the coil held at ≍30 cm of the head) were rectified and averaged, and then RT was measured visually where EMG first rose above baseline activity (Hodges and Bui, 1996). The RT corresponds to the delay between the Go signal and the EMG onset of LES (Rowland et al., 2021). RT was calculated for each participant at the beginning of the experiment and used to time the TMS and SR stimulations in the execution period.

A total of 45 stimulations were applied for each of the five paradigms tested [PA-TMS (single- and paired-pulse), AP-TMS (single- and paired-pulse), and SR]. For each paradigm, a total of 15 stimulations per condition were applied. Fifteen trials were selected as the best compromise to test all our conditions without inducing too much fatigue by a long experimentation. The average amplitude of 10 MEPs has been proved to provide excellent test–retest reliability (Cavaleri et al., 2017a). Conditions were distributed in three blocks of five stimulations. The testing order was randomized at the levels: (1) neurophysiological techniques (PA-TMS, AP-TMS, SR), (2) TMS paradigms (single-, paired-pulse—for TMS only), and (3) conditions (preparation period, execution period, and baseline; Fig. 1*D*). The randomization process was repeated for each neurophysiological technique and TMS paradigm. Randomization orders were generated using the Excel function “*RANDBETWEEN*” for the neurophysiological techniques and TMS paradigms. A custom script in Spike2 (Cambridge Electronic Design) automatically and randomly generated the order of the conditions for each block of stimulation and the interval between each condition (between 7 and 10 s). Randomization is critical to ensure that differences detected were due to the different conditions rather than an effect of time/order. Participants were asked to rest between blocks to limit fatigue.

### Electromyography activity recording and MVC

Surface electromyography (EMG) signals were recorded from the right LES with pairs of surface Ag/AgCl electrodes (Kendall Medi-trace 200, Covidien) positioned on the muscle belly of the LES at L3–L4 level following SENIAM recommendations (Hermens et al., 2000). The ground electrode used was large (9160F, 3M) and was positioned over both the right anterosuperior iliac spine and iliac crest (anterior and medial part) to reduce TMS artifact. EMG signals were amplified (×1,000), bandpass filtered between 10 and 500 Hz using a D360 EMG amplifier (Digitimer), and digitized at a sampling rate of 1,000 Hz using a CED Power1401 Data Acquisition System with Spike2 software.

Maximal resisted trunk extension and maximal pelvic tilt were evaluated once for each participant at the beginning of the experimentation (Desmons et al., 2021). The task that produced the largest LES EMG activity was performed three more times to determine the MVC. Participants performed three 3 s maximal resisted trunk extensions or maximal pelvic anteversion in sitting, and verbal encouragements were provided by the experimenters. Breaks of ∼45 s were taken between trials. The maximal peak value obtained over the three trials was considered as MVC [root mean square (RMS) EMG activity over a 100 ms window around the peak amplitude]. The MVC was used to set up the background EMG activity used during the session. Following MVC (but before any neurophysiological measurements), participants were tested regarding their ability to activate LES at 15 ± 5% MVC. For two participants, the LES EMG activity exceeded the 15% MVC level even though LES were relaxed (i.e., while flexing the low back spine in sitting) or when they were asked to sit upright. This was due to the small absolute EMG amplitude during MVC. Considering it would have been very difficult to elicit MEPs with small or no LES activation, we decided to adjust the relative level of LES contraction for these participants (Rohel et al., 2022). The level of contraction requested was increased by steps of 5% until participants could maintain an upright sitting position with a slight lumbar lordosis. Once the adjustment was done, the EMG was kept constant for the whole session.

### Transcranial magnetic stimulation

A monophasic Magstim BiStim^2^ stimulator (The Magstim) connected to a double-cone coil (126 mm diameter per wing; Magstim) was used to deliver TMS. Coil orientation and position was guided with a stereotaxic neuro-navigation system (Brainsight, Rogue Research).

Since there is no evidence that handedness influences TMS outcomes for back muscles (Nowicky et al., 2001; O’Connell et al., 2013, Massé-Alarie et al., 2016a, 2018; Chiou and Strutton, 2020), all TMS paradigms were performed over the left hemisphere. The optimal stimulation site on the scalp (hotspot) was defined as the location that elicits the largest MEP amplitude at a given intensity in the contralateral LES (Groppa et al., 2012). The active motor threshold (AMT) corresponded to the lowest stimulation intensity required to evoke at least three MEPs out of six stimulations at a physiological latency (∼12–20 ms), which were clearly discernible from background EMG activity (Taniguchi and Tani, 1999; Strutton et al., 2005; Desmons et al., 2021). TMS pulses were triggered automatically at a random interval between 7 and 10 s for every trial of stimulation using a custom-made script in Spike2. Hotspot and AMT were identified for each current direction (PA- and AP-TMS) during a pre-activation of LES at 15 ± 5% MVC. TMS methods were reported and controlled according to the TMS-specific checklist [Chipchase, 2012; Extended data 1 (https://doi.org/10.5683/SP3/RE08GP)].

Single-pulse stimulation intensity was adjusted to 120% AMT. SICI paradigm was tested using TMS paired pulse (Kujirai et al., 1993). Conditioning stimulus was set at 80% AMT and the test stimulus at 120% AMT with an interstimulus intervals of 3 ms; these parameters have been shown to optimize SICI of LES M1 representation in both current directions (Desmons et al., 2021). All stimulations (single-pulse stimulation, paired-pulse conditioning, and test stimulation) were applied on the hotspot corresponding to the current direction tested. Considering that testing multiple muscles would have complexified the study design (e.g., different EMG activation in a task, different hotspots, different AMT), only LES was tested.

### Stretch reflex (muscle tap)

A homemade electromagnetic hammer device was designed and built to elicit SR. The hammer was powered by a 24 V solenoid and controlled via a homemade script on Spike2 software and a CED Power1401. A spring ensures the impinger to return and keep the duration of the impact as short as possible since the tap duration may influence the SR latency (Skotte et al., 2005). The system used in this study produced a tap duration of ∼8 ms, which is similar to previous study [∼4.2 ms (Skotte et al., 2005) and ∼12.5 ms (Zedka et al., 1999)]. A force sensor (FlexiForce A201, Tekscan) located at the end of the impinger indicated the moment of contact allowing to measure SR latency. Muscle taps were applied ∼1–2 cm medial and cranial to the right posterosuperior inferior spine (PSIS), at the maximal force of the hammer (Rohel et al., 2022). The SR is composed of a short latency (R1) ∼12–22 ms (Tani et al., 1997; Zedka et al., 1999; Skotte et al., 2005) and a longer latency (R2) ∼35–50 ms (Tani et al., 1997; Zedka et al., 1999) responses. R1 would represent the excitability of a monosynaptic spinal loop elicited by the depolarization of the primary spindle afferent by the muscle tap, whereas the underlying neuronal circuits of R2 remains mostly unknown (Marsden et al., 1976) but would likely represent the excitability of a supraspinal loop (Day et al., 1991). Only R1 amplitude was measured to estimate spinal excitability.

### Data analysis

#### Data cleaning

All trials were visually checked to remove false starts using a two-step process: (1) when LES was activated before the Go signal and (2) using Grubbs’ tests for outlier detection to ensure that nonphysiological RT values did not influence the results (Grubbs, 1969). On average, 14.6/15 trials were available for each condition. Each single MEP was visually checked to ensure it did not fall into the LES EMG burst.

#### Motor-evoked potentials

Using an off-line custom-made MATLAB script, each TMS pulse was displayed for visual identification of peak-to-peak MEP amplitude. The experimenter performed the analyses without being aware of the current direction. Single-pulse MEP amplitudes measured in preparation and execution periods were expressed as a percentage of change from the average baseline MEP amplitude:

$$=\frac{\left(\mathrm{average}\;\;\mathrm{raw}\;\mathrm{MEP}\;\mathrm{amplitude}\;\mathrm{during}\;\mathrm{preparation}/\mathrm{execution}\;\mathrm{periods}-\mathrm{average}\;\mathrm{raw}\;\mathrm{MEP}\;\mathrm{amplitude}\;\mathrm{at}\;\mathrm{baseline}\right)}{\left(\mathrm{average}\;\mathrm{raw}\;\mathrm{MEP}\;\mathrm{amplitude}\;\mathrm{at}\;\mathrm{baseline}\right)}\;x\;100.$$

Conditioned MEP amplitude was expressed, as a percentage of the MEP test amplitude measured in the same interval period (preparation or execution).

$$=\frac{\mathrm{conditioned}\;\mathrm{MEP}\;\mathrm{amplitude}\;\mathrm{during}/\mathrm{execution}\;\mathrm{period}\;}{\mathrm{test}\;\mathrm{MEP}\;\mathrm{amplitude}\;\mathrm{during}\;\mathrm{preparation}/\mathrm{execution}\;\mathrm{period}}\;x\;100.$$

Then, differences between the conditioned MEP amplitude (in percentage of the test MEP) measured during preparation/execution periods and the baseline were calculated to measure the SICI change at the different intervals.

=conditonedMEP(%test)duringpreparation/execution-conditonedMEP(%test)atbaseline.

#### Short latency stretch reflex (R1)

Using an off-line custom-made script in MATLAB, R1 signal was bandpass filtered (70–500 Hz) to reduce the EMG noise elicited by the muscle tap realized at the vicinity of the recording electrodes. Similar techniques were used for limiting the impact of peripheral stimulations (electrical, magnetic) on EMG of lumbar back muscles (Massé-Alarie et al., 2019, 2022). Considering the identification of individual R1 (elicited by a single muscle tap) may be difficult in some participants because of the small signal-to-noise ratio, the average of 15 EMG traces per condition was used to ease the identification of the motor response (Dimitrijevic et al., 1980). For postural task, R1 was identified in 12/15 participants for baseline, 10/15 for the preparation period, and 12/15 for the execution period. For voluntary task, R1 was identified in 12/15 participants for baseline, 11/15 for the preparation period, and 13/15 for the execution period. A time window was used to measure EMG peak-to-peak for participants for whom R1 was not visually identified (Desgagnes et al., 2021). The time window duration was calculated using the average R1 onset and offset for all participants for whom a motor response was identified [12 ms (onset) to 27 ms (offset) following the muscle tap]. R1 amplitude during preparation/execution periods were expressed in percentage of R1 amplitude measured at baseline.

#### EMG during motor tasks

Off-line data analysis of raw EMG signals was done using a custom-made MATLAB script (The MathWorks). The rectified EMG amplitude of 10 trials from the RT block without TMS or muscle taps was calculated and divided into sixty 50 ms epochs starting at the 500 ms prior to the Warning signal and ending 1,500 ms after the Go signal. The epoch with the highest average rectified EMG amplitude was identified for each motor task for further analysis. The objective of this analysis was to determine if potential differences in MEP changes could be explained by upcoming changes in EMG activity associated with the motor tasks.

### Statistical analysis

Normal distribution of the data was tested using the Shapiro–Wilk's test. Since most of data did not follow a normal distribution and no transformation normalized the distribution, nonparametric tests were used. For multidimensional analyses, nonparametric analyses of variance for longitudinal data (nparLD) were used. nparLD is a robust method for factorial designs with small and inequivalent samples; it is also robust with missing data and outliers and does not require normality of distributions and homoscedasticity (Noguchi et al., 2012). Post hoc tests were done using the one-way nparLD (nparLD package; Karch, 2021) and adjusted for multiple comparisons using false discovery rate methods (Benjamini and Hochberg, 1995). ANOVA equivalent *F* test for nparLD is reported as ANOVA type statistics (ATS) with degree of freedom (df) in the Results section. Relative treatment effect (RTE) from nparLD analysis is reported as an indicator of the effect size in Table 2. Cutoff values for the interpretation of the effect size statistics RTE have been proposed: 0.56 (small), 0.64 (medium), and 0.71 (large; Vargha and Delaney, 2000). *p* values of the nParLD were bootstrapped (5,000 iterations) to obtain an “observed power” representing the proportion of significant tests (*α* = 0.05). For the subset analysis, only 90 iterations were used since higher number of iterations did not converge. Observed powers are reported in Table 2. All statistical analyses were performed with the R Studio (version 1.3.1093, Rstudio: Integrated Development Environment for R, PBC).

**Table 2.** Statistical table

Analysis	*n*	Figure	Type of test	Effect sizes for main effects (RTE)	Observed power	Post hoc analysis	Effect size for significant interaction (RTE)	Observed power for significant interaction
Main objective: postural task								
Normalized MEP amplitude: current direction × period (preparation and execution)	15	Figures 4*a* and 5*a*; Tables 3 and 4	Two-way nparLD	Current: 0.51 Period: 0.64*	Current direction: 0.18 Period: 0.97* Interaction: 0.21			
Normalized SICI: current direction × period	15	Figures 4*b* and 5*b*; Tables 3 and 4	Two-way nparLD	Current: 0.53 Period: 0.54	Current direction: 0.08 Period: 0.12 Interaction: 0.10			
Normalized R1 amplitude: period	15	Figures 4*a* and 5*a*; Tables 3 and 4	One-way nparLD	Period: 0.60*	Period: 0.691*			
Main objective: voluntary task								
Normalized MEP amplitude: current direction × period (preparation and execution)	13	Figures 4*a* and 5*a*; Tables 3 and 4	Two-way nparLD	Current: 0.54	Current direction: 0.37 Period: 0.59* Interaction: 0.61*	PA (Prep. Exe)	0.54	0.16
						AP (Prep. Exe)	0.65*	0.84*
				Period: 0.60*		Prep (PA. AP)	0.55	0.33
						Exe (PA. AP)	0.61*	0.60*
Normalized SICI: current direction × period	13	Figures 4*b* and 5*b*; Tables 3 and 4	Two-way nparLD	Current: 0.53	Current direction: 0.11 Period: 0.09 Interaction: 1.00*	PA (Prep. Exe)	0.62*	0.83*
						AP (Prep. Exe)	0.56	0.34
				Period: 0.52		Prep (PA. AP)	0.62	0.46
						Exe (PA. AP)	0.55	0.19
Normalized R1 amplitude: period	13	Figures 4*a* and 5*a*; Tables 3 and 4	One-way nparLD	Period: 0.59*	Period: 0.536*			

Limits for interpretation: 0.56 (small), 0.64 (medium), 0.71 (large; Vargha and Delaney, 2000). AP, anteroposterior; normalized MEP, motor-evoked potential (in percentage of baseline MEP); PA, posteroanterior; normalized R1, short latency response (in percentage of baseline R1); RTE, relative treatment effect; normalized SICI, short latency intracortical inhibition (in percentage of baseline); nparLD, nonparametric longitudinal data analysis; Prep, preparation; Exec, execution.

* Statistics associated with a *p* value <0.05.

*Preliminary analyses not related to study objectives*: motor thresholds and MEP latencies measured for the different current directions were compared using one-way nparLD.

#### Main objective

To determine if differences in the change of corticospinal, spinal excitability, and intracortical inhibition occurred between current directions, the following statistical analyses were computed, independently for each experiment:

1. Corticospinal excitability (MEP amplitude % change from baseline), using factors current direction (PA- and AP-TMS) and period [preparation and execution (two-way nparLD)].
2. Inhibitory (SICI) intracortical circuits (conditioned MEP amplitude, difference with baseline), using factors current direction and period (two-way nparLD).
3. Motoneuronal excitability (R1 amplitude % change from baseline) using period (one-way nparLD).

#### Secondary objectives

1. To determine if significant change of our variables occurred during the preparation and execution periods when compared with baseline values, we computed a one-way nparLD comparing MEP amplitude, SICI (% test), or R1 amplitude average raw values during preparation and execution periods to their corresponding baseline values for both tasks, for each interval separately.
2. To determine if different change of our variables occurred between the postural and voluntary tasks, we performed statistical tests on the subset of participants (*n* = 6) who performed both experiments; the following statistical analyses were computed, independently for each period:
  Corticospinal excitability, using factors current direction and task (postural vs voluntary tasks; two-way nparLD).
  Inhibitory (SICI) intracortical circuits, using factors current direction and task (two-way nparLD).
  Motoneuronal excitability using factor task (one-way nparLD).

To ensure that potential differences between tasks were not driven by larger EMG activation elicited by the motor tasks, we compared the highest EMG amplitude of the 50 ms epoch of EMG during RT measurement between task using a one-way nparLD.

The median (interquartile range) is reported throughout the text and figures unless otherwise specified.

## Results

Fifteen participants performed the postural task experiment [age, 30.5 (5.7) years; 13 right-handed; 8 women], and 15 participants performed the voluntary task although two were excluded due to technical issues during data collection. Thus, 13 participants are included in the voluntary task experiment analysis [age, 30.1 (5.3) years; 11 right-handed; 7 women]. For two participants, it was difficult to evoke consistent MEPs at 15% MVC; thus 30% MVC was used throughout the session. Nine participants undertook the two experiments but data from six participants were available [age, 32.3 (6.0) years; 5 right-handed; 4 women]; two participants were excluded because of the technical issue reported, and one was excluded since a different percentage of MVC was used in the different tasks. Characteristics of each group are described in Table 1.

### Hotspot

Extended data 2 (https://doi.org/10.5683/SP3/RE08GP) reported the hotspot coordinate for PA- and AP-TMS for the postural and voluntary tasks in hotspots in MNI coordinate system (Montreal Neurological Institute and Hospital).

### Latency

MEP latencies were longer with AP-TMS than those with PA-TMS [Experiment 1—PA, 15.91 (2.37) ms; AP, 16.22 (2.07) ms; ATS = 4.12; df = 1; *p* < 0.004 | Experiment 2—PA, 15.45 (1.31) ms; AP, 16.17 (1.89) ms; ATS = 6.75; df = 1; *p* < 0.0093).

### Active motor threshold

A higher TMS output was needed to obtain AMT in AP- compared with PA-TMS for both experiments [Experiment 1—PA, 56.0 (10.5) %; AP, 65.0 (13.7) % MSO; *p* < 0.001 | Experiment 2—PA, 54.0 (12.0) %; AP, 61.0 (17.2) % MSO; *p* = 0.005].

### Description of MEP, R1, and SICI EMG traces

**Figure 2.**
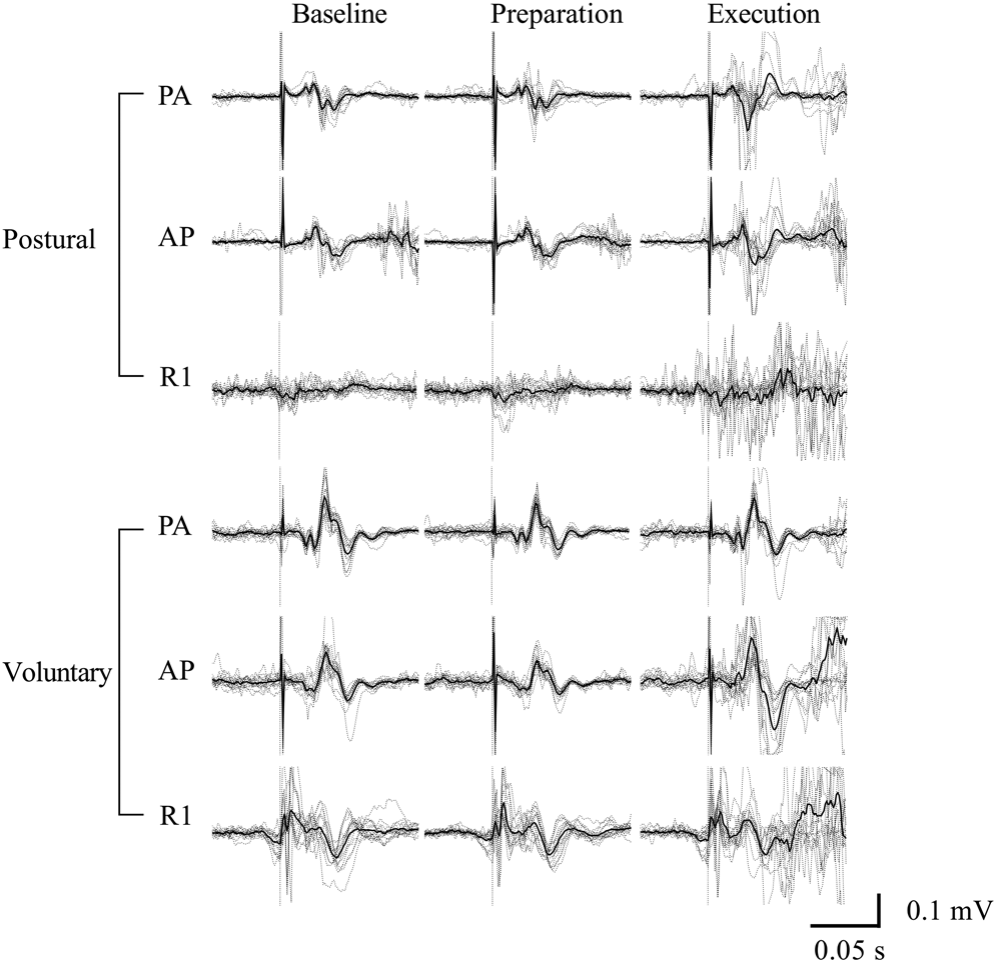
Examples of motor responses elicited by single-pulse PA-TMS, AP-TMS, and short latency response (R1) of the stretch reflex evoked by a muscle tap observed for the different conditions (baseline, preparation, and execution) and tasks for one participant. The bold line represents the average signal; dotted lines represent individual EMG traces (*n* = 15), and vertical dotted lines represent the stimulation. AP, anteroposterior; PA, posteroanterior; R1, short latency response.

**Figure 3.**
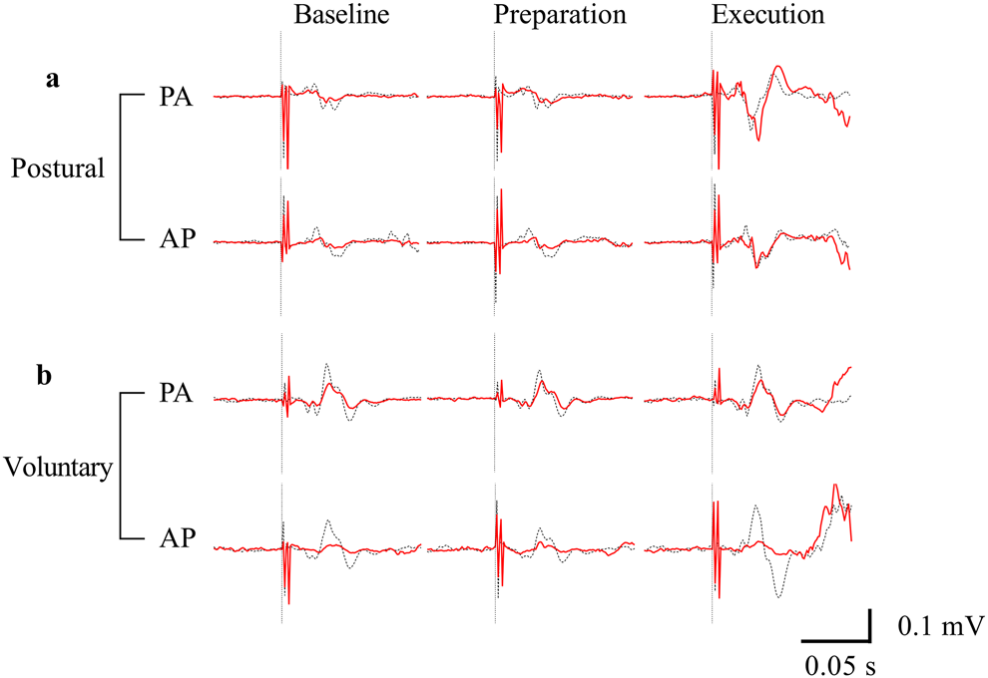
Examples of motor responses elicited by paired-pulse PA-TMS and AP-TMS observed for the different conditions (baseline, preparation, and execution) and tasks for one participant. The red bold line represents the average signal of the paired-pulse conditioned MEP; the black dotted line represents average signal of the single-pulse test MEP, and vertical dotted lines represent the conditioning stimulation for the paired-pulse stimulations and the test stimulus for the single-pulse stimulations. AP, anteroposterior; PA, posteroanterior; R1, short latency response.

Figures 2 and 3 are examples of raw EMG signals [MEPs and R1 (Fig. 2) and SICI (Fig. 3)].

### Experiment 1: bilateral shoulder flexion—postural task

**Figure 4.**
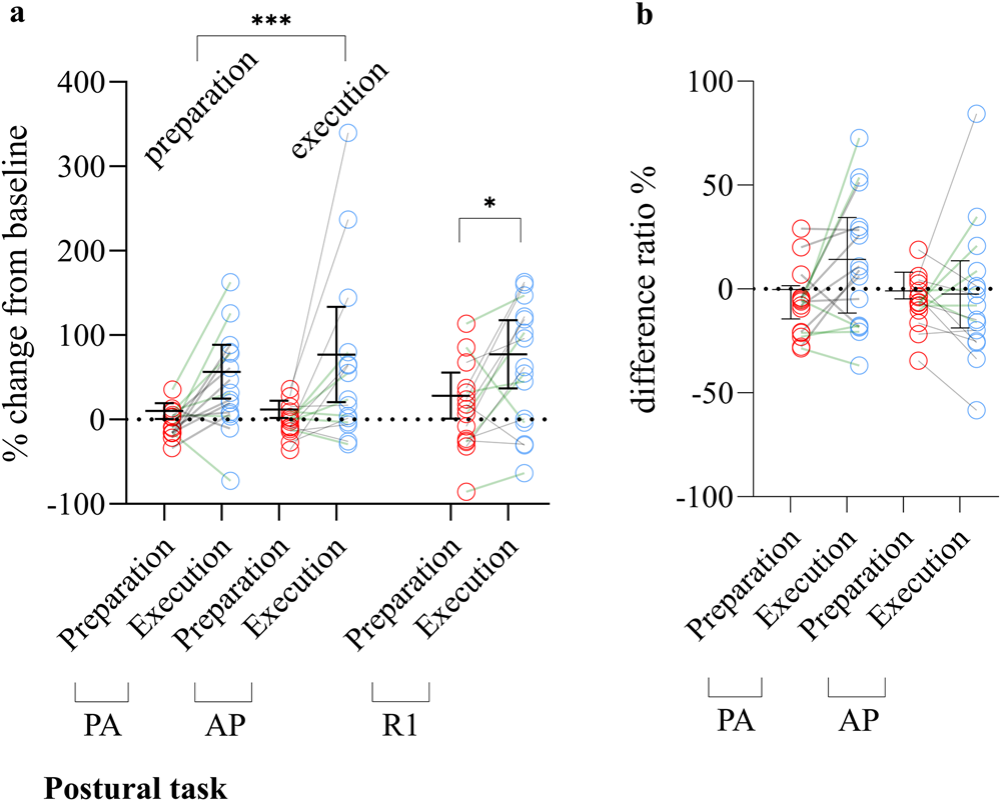
Comparison of the modulation of MEP, R1, and SICI in preparation and execution of postural task for both current directions. ***a***, MEP and R1 modulation are expressed as the percentage of change relative to baseline. A main effect of the period on the change of MEP amplitude regardless of the current direction was observed (*p* = 0.0002—depicted as the comparison between preparation and execution labels in Fig. 4*a*): R1 amplitude was also larger in the execution compared with that in preparation (*p* = 0.02). ***b***, SICI modulation is expressed as the difference between SICI ratio in the preparation/execution period with the baseline. Error bars correspond to interquartile range. Orange lines between circles represent participants from the subset, and gray lines between circles represent the participants that only took part in Experiment 1. AP, anteroposterior; PA, posteroanterior; R1, short latency response; MEP, motor-evoked potential; TMS, transcranial magnetic stimulation. *p < 0.05; ***p < 0.001.

Figure 4 displays results referring to the main objectives and the associated analyses. Table 3 presents results related to the secondary objective (1) for the postural task.

**Table 3.** Changes of MEP and R1 amplitudes using single- and paired-pulse TMS during different periods of movement preparation and execution of the postural task

Variable	Baseline	Preparation		Execution	
	Amplitude, mV	Amplitude, mV	*p* value difference from baseline	Amplitude, mV	*p* value difference from baseline
MEP-PA	0.114 (0.065)	0.134 (0.101)	0.10	0.184 (0.153)	**<0.0001**
MEP-AP	0.108 (0.095)	0.114 (0.081)	**0.002**	0.184 (0.163)	**0.0001**
R1	0.015 (0.013)	0.019 (0.023)	**0.02**	0.020 (0.024)	**0.001**
	Cond MEP, % MEP test	Cond MEP, % MEP test		Cond MEP, % MEP test	
SICI-PA	69.363 (34.067)	73.375 (25.121)	0.92	82.696 (32.173)	**0.03**
SICI-AP	73.19 (31.38)	71.75 (34.79)	0.83	78.39 (31.09)	0.77

AP, anteroposterior; Cond MEP, conditioned motor-evoked potential; MEP, motor-evoked potential; MEP test, test motor-evoked potential; PA, posteroanterior; R1, short latency response; SICI, short-interval intracortical inhibition; TMS, transcranial magnetic stimulation. % MEP test: percentage of the amplitude of the MEP test. Significative p-value are indicated in bold.

#### MEP amplitude

The change in MEP amplitude regardless of current direction was higher in the execution [40.5 (77.3) % baseline] compared with that in the preparation [7.7 (20.5) % baseline | main effect, period; ATS = 14.33; df = 1; *p* = 0.00015; observed power = 0.97; Fig. 4*a*, left panel]. No other main effect or interaction was observed.

#### R1 amplitude

R1 change was significantly larger in the execution period [77.0 (124.5) % baseline] compared with that in preparation [28.1 (61.3) % baseline—ATS = 5.74; df = 1; *p* = 0.017; observed power = 0.69; Fig. 4*a*, right panel].

#### SICI

Figure 4*b* illustrates the difference in SICI within preparation and execution periods. No significant main effect or interaction was present.

Table 3 reports the change in MEP and R1 amplitudes using single- and paired-pulse TMS in the motor preparation and execution periods. During the preparation period, MEP amplitude elicited by AP-TMS (*p* = 0.0019) and R1 (*p* = 0.015) were significantly increased compared with baseline. During the execution period, PA-MEP (*p* < 0.0001), AP-MEP (*p* = 0.00014), PA-SICI (*p* = 0.032), and R1 (*p* = 0.00083) were significantly higher compared with baseline values (Table 3). Extended data 3 (https://doi.org/10.5683/SP3/RE08GP) reported the individual raw amplitudes of single- and paired-pulse MEP and R1.

### Experiment 2: anterior pelvic tilt—voluntary task

**Figure 5.**
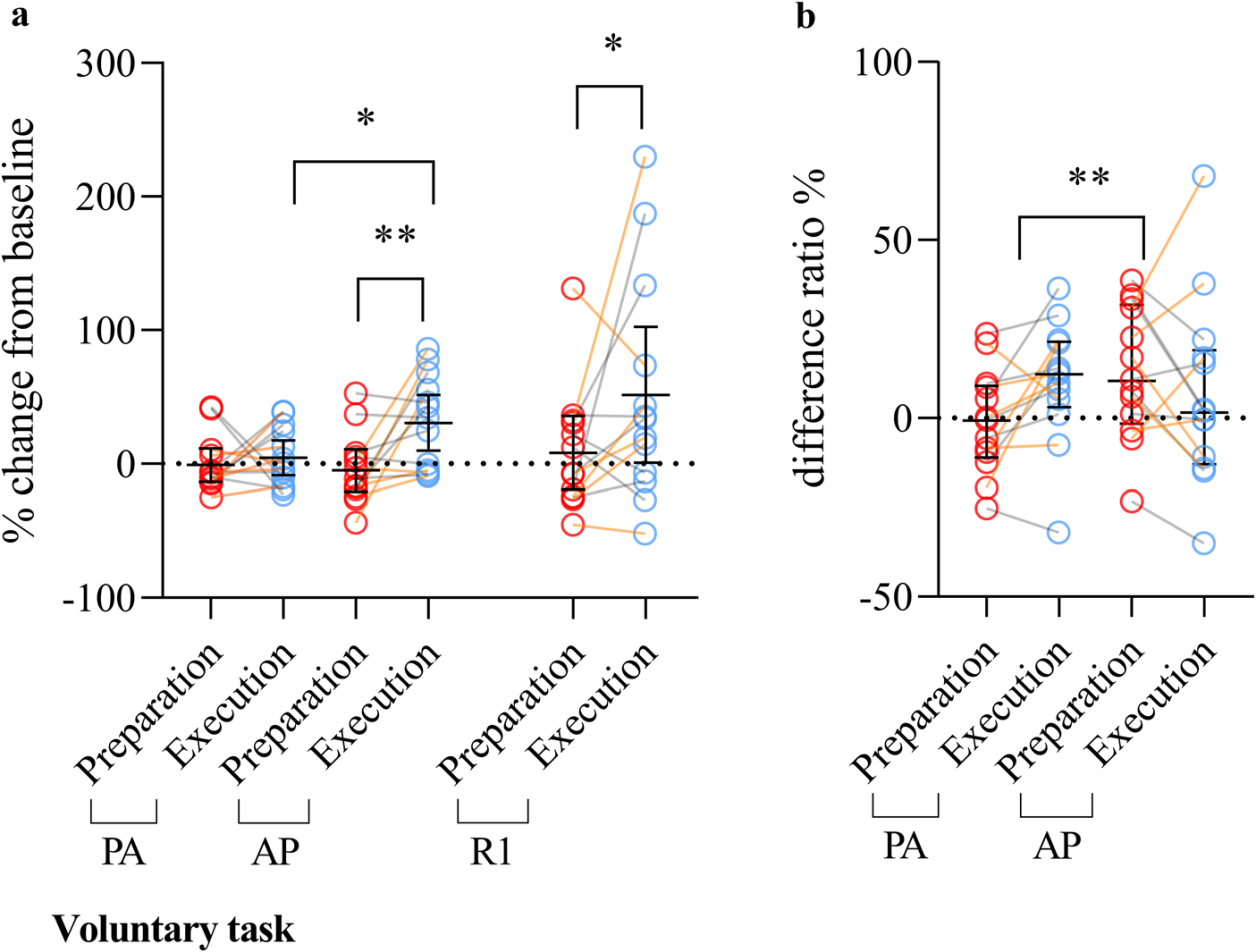
Comparison of the change of MEP, R1, and SICI in preparation and execution of the voluntary task for both current directions. ***a***, MEP and R1 modulation are expressed as the percentage of change relative to baseline. Significant period × current interaction was observed (*p* = 0.03). A larger change of AP-MEP amplitude was present in the execution compared with that in the preparation period (*p* = 0.016). R1 amplitude was larger in the execution compared with that in the preparation period (*p* = 0.03). ***b***, SICI modulation is expressed as the difference between SICI ratio in the preparation/execution period with the baseline. Significant period × current interaction was observed (*p* = 0.0003). PA-SICI difference was smaller in preparation in comparison with that in the execution period (*p* = 0.028). Error bars correspond to interquartile range. Orange lines between circles represent participants from the subset, and gray lines between circles represent the participants that only took part in Experiment 2. AP, anteroposterior; PA, posteroanterior; R1, short latency response; MEP, motor-evoked potential; TMS, transcranial magnetic stimulation. *p < 0.05; **p < 0.01.

Figure 5 depicts results referring to the main objectives and the associated analyses. Table 4 presents results related to the secondary objective (1) for the voluntary task.

**Table 4.** Changes of MEP and R1 amplitudes using single- and paired-pulse TMS during different periods of movement preparation and execution of the voluntary task

Variable	Baseline	Preparation		Execution	
	Amplitude, mV	Amplitude, mV	*p* value difference from baseline	Amplitude, mV	*p* value difference from baseline
MEP-PA	0.129 (0.092)	0.140 (0.086)	0.67	0.126 (0.139)	0.81
MEP-AP	0.112 (0.056)	0.099 (0.060)	0.21	0.152 (0.126)	**0.02**
R1	0.012 (0.017)	0.017 (0.018)	0.87	0.024 (0.023)	0.08
	Cond MEP, % MEP test	Cond MEP, % MEP test		Cond MEP, % MEP test	
SICI-PA	63.398 (18.630)	65.929 (22.246)	0.58	77.799 (16.505)	**0.048**
SICI-AP	58.901 (26.065)	76.326 (38.773)	0.15	65.217 (28.586)	0.54

AP, anteroposterior; Cond MEP, conditioned motor-evoked potential; MEP, motor-evoked potential; MEP test, test motor-evoked potential; PA, posteroanterior; R1, short latency response; SICI, short latency interval intracortical inhibition; TMS, transcranial magnetic stimulation. % MEP test: percentage of the amplitude of the MEP test. Significative p-value are indicated in bold.

#### MEP amplitude

A significant period × current interaction was observed (*F* = 4.74; *p* = 0.029). This interaction was explained by a larger change in AP-MEP amplitude presence in execution [33.8 (62.3) % baseline] compared with preparation [−11.6 (28.4) % baseline—ATS = 8.28; df = 1; *p* = 0.016; observed power = 0.84; Fig. 5*a*]. In the execution period, AP-MEP amplitude change was larger than PA-MEP [−1.0 (42.3) % baseline—ATS = 4.83; df = 1; *p* = 0.056; observed power = 0.60; Fig. 5*a*]. No difference was present for other pairwise comparisons.

#### R1 amplitude

R1 change was significantly larger in the execution [33.9 (105.0) % R1 test] compared with that in preparation period [−7.4 (55.5) % R1 test—ATS = 4.51; df = 1; *p* = 0.034; observed power = 0.54; Fig. 5*a*].

#### SICI

A significant period × current interaction was observed (ATS = 13.12; df = 1, *p* = 0.0003). PA-SICI difference was smaller in preparation [−0.7 (19.9) %] in comparison with that in the execution period [9.3 (22.2) %—ATS = 7.28; df = 1; *p* = 0.028; observed power = 0.83; Fig. 5*b*]. No other difference was observed. Table 4 reports the change in MEP and R1 amplitude using single- and paired-pulse TMS in the preparation and execution periods of the voluntary task. During the execution period, AP-TMS (*p* = 0.021) and PA-SICI (*p* = 0.025) were significantly higher compared with baseline values. Extended data 3 (https://doi.org/10.5683/SP3/RE08GP) reported the individual raw amplitudes of single- and paired-pulse MEP and R1.

### Secondary analyses: motor task comparisons in a subset of participants (*n* = 6)

**Figure 6.**
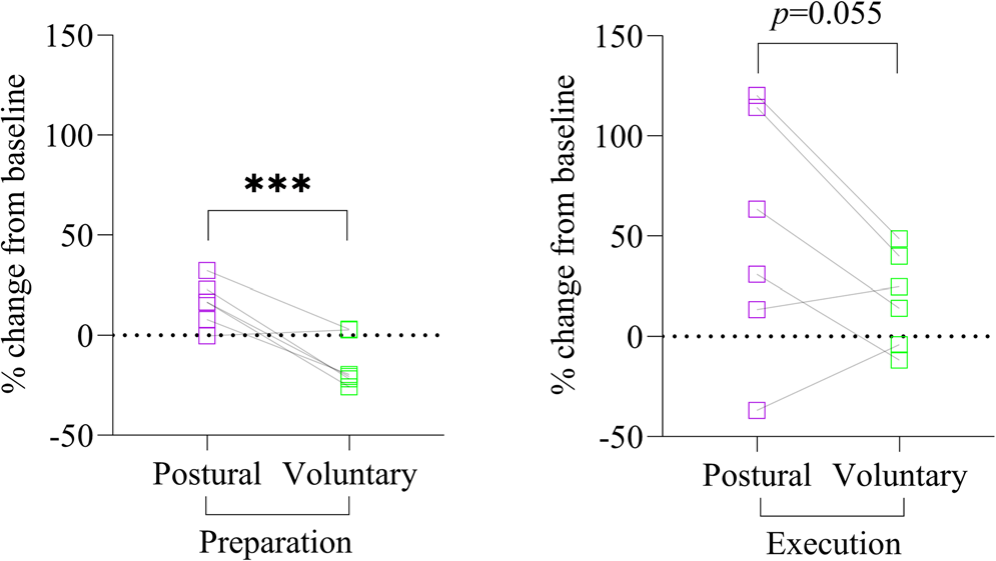
Individual normalized MEP amplitudes of the subset of participants (*n* = 6) expressed as percentage of MEP test from baseline. This subset of participants performed both experiments to determine if different types of tasks (postural vs voluntary) differently impacted neural circuits excitability change. PA- and AP-TMS were averaged in each period (preparation and execution). MEP amplitude change (regardless of the current direction) was larger in the postural task compared with that in the voluntary task (*p* < 0.0001) for the preparation period and in the execution period (*p* = 0.055). AP, anteroposterior; PA, posteroanterior; MEP, motor-evoked potentials; TMS, transcranial magnetic stimulation. ***p < 0.0001.

Figure 6 illustrates the change of single-pulse MEP and R1 amplitude between postural and voluntary tasks. The change in MEP amplitude regardless of the current direction was higher in the postural task [16.5 (19.4) % baseline] compared with that in the voluntary task [−20.0 (25.5) % baseline | main effect, task; ATS = 16.13; df = 1; *p* < 0.0001] for the preparation period. In addition, the change in MEP amplitude regardless of the task was higher with PA-TMS [3.6 (13.0) % baseline] compared with that with AP-TMS [−4.2 (13.4) % baseline | main effect, task; ATS = 9.87; df = 1; *p* = 0.002; Fig. 6*a*). Finally, in the execution period, the change in MEP amplitude regardless of the current direction just missed the alpha threshold (main effect, task; ATS = 3.67; df = 1; *p* = 0.055): postural task [47.2 (114.9) % baseline] and voluntary task [19.4 (48.1) % baseline; Fig. 6*b*]. No other main effect or interaction was significant.

### EMG

**Figure 7.**
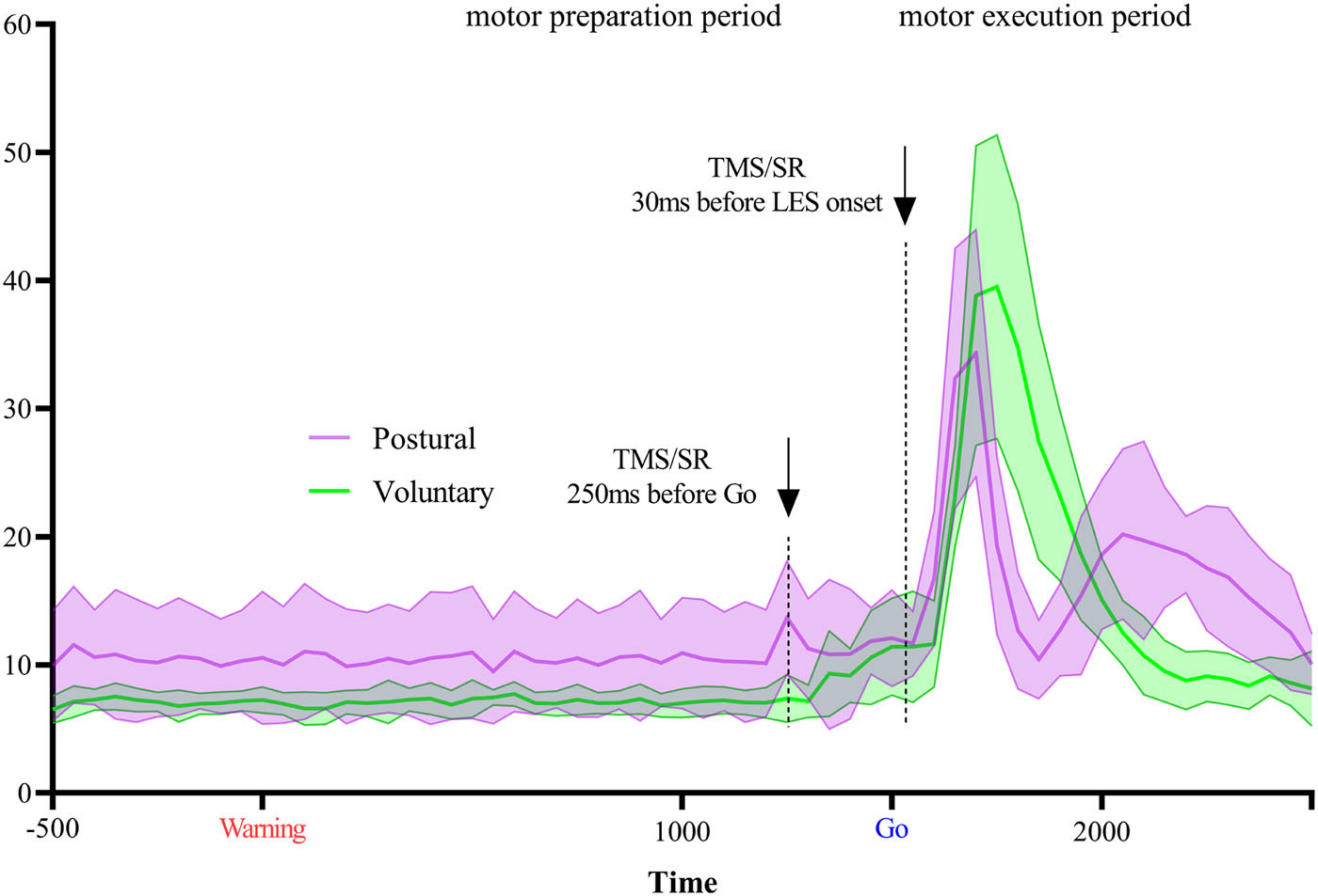
Rectified and averaged EMG signal (over 10 trials) of the subset (*n* = 6) of participants who participated in both experiments expressed as percentage of MVC. The bold lines represent the mean signal, whereas the lighter envelopes represent the standard deviation. The traces represent the rectified EMG amplitude from the RT block without TMS or muscle taps, corresponding to sixty 50 ms epochs starting 500 ms before the Warning signal and ending 1,500 ms after the Go signal. The average maximal EMG amplitude was larger during the postural task compared with that during the voluntary task (*p* = 0.005). EMG, electromyography; MVC, maximal voluntary contraction; TMS, transcranial magnetic stimulation; SR, stretch reflex.

Figure 7 illustrates the average rectified EMG during the preparation and the motor execution periods for the subset of participants (*n* = 6). A larger average maximal EMG amplitude elicited by postural task [41.7 (26.4) % MVC] compared with voluntary task [63.7 (49.3) % MVC—ATS = 7.86; *p* = 0.005] was observed.

## Discussion

These series of experiments investigated the contribution of various neural circuits interacting with TMS involved in the control of low back muscles during the preparation and execution of two different dynamic motor tasks. The tasks were selected to investigate the LES in different functional roles, that is, (1) postural control of the trunk during upper limb movement (“*prime controller*”) or (2) voluntary control as the *prime mover* of the lumbar spine in extension. Results did not support initial hypotheses. In the postural task, corticospinal and motoneuronal excitability changes were higher during the execution than preparation regardless of the current direction. In the voluntary task, AP-MEP and R1 changes were higher during the execution than those during the preparation, although no change was present using PA-TMS. The fact that PA- and AP-TMS MEP amplitude were influenced differently depending on the tasks suggests that the contribution of neural circuits may depend on the LES role. Although it could be argued that TMS is not sensitive enough to measure change in MEP amplitude in the voluntary task, the fact that (1) PA-MEP was significantly increased during the postural task and (2) AP-MEP was significantly increased in the voluntary task in execution compared with that in preparation strongly suggests that a change in the neural circuits tested should have been measured if present. Our secondary analyses of the subset (comparisons between tasks) suggest a larger increase in corticospinal excitability in postural than that in voluntary tasks, implying a smaller contribution of the neural circuits tested by TMS [e.g., cortical and spinal circuits, corticospinal tract (Siebner et al., 2022)] in the voluntary task for the preparation and perhaps the execution periods.

### PA-TMS and PA-TMS sensitive circuits involved in postural and voluntary control of LES

It is important to note that although some evidence (discussed in the next section) point toward the recruitment of neurons in the premotor/SMA using AP-TMS, change in AP-MEP could also reflect partially the M1 state since premotor cortex and SMA both project to (Nachev et al., 2008; Neige et al., 2021) and may influence M1 excitability (Reis et al., 2008). Similarly, it is important to consider that TMS was applied over the hotspot that was mostly positioned over M1 [based on MNI coordinates—Extended data 2 (https://doi.org/10.5683/SP3/RE08GP)] for both current directions, and then, the study design does not allow to determine precisely which cortical areas were depolarized by AP-TMS and PA-TMS. Although underlying mechanisms remain speculative and based on indirect evidence, the following elements support the hypothesis that different current directions interact with distinct neuronal circuits. First, we replicated results of higher AMT and longer MEP latency elicited by AP- compared with PA-TMS for LES (Desmons et al., 2021). Second, for the voluntary task, a larger change in the excitability of corticospinal projections to LES was observed for AP-TMS in execution compared with that in preparation which was not the case for PA-TMS. Based on the general hypothesis that AP-TMS recruits premotor/SMA circuits and PA-TMS recruits M1 (Di Lazzaro and Rothwell, 2014; Aberra et al., 2020), our results suggest, against our initial hypothesis, a larger involvement of premotor/SMA regions than M1 in the control of LES as a *prime mover* of the lumbar spine. In contrast, for the postural task, no difference between current directions was observed (same increase in excitability) suggesting a similar implication for both circuits. Furthermore, in the subset analysis, PA-MEP amplitude was higher than AP-MEP during the preparation period regardless of the task, indicating a difference in the effect of current direction between tasks. Third, PA-SICI was reduced during the execution period for both tasks, whereas no change was present for AP-SICI. For distal muscles, inhibition elicited by PA-SICI protocol is usually released prior to the movement (Reynolds and Ashby, 1999; Nikolova et al., 2006) although the change of AP-SICI using a RT paradigm remains unexplored. SICI is known to represent the excitability of local γ-aminobutyric acid (GABA)ergic inhibitory circuits in M1 (Kujirai et al., 1993; Hanajima et al., 1998). GABA_A_ interneurons appear to play a role in the selection process, disinhibiting neural circuits possibly to drive activity toward the critical triggering threshold for depolarization (Derosiere and Duque, 2020). Our results suggest a specific inhibition with PA-SICI protocol for motor execution but not with AP-SICI protocol. However, the release in inhibition with PA-SICI in absence of a PA-MEP amplitude increase during the execution of the voluntary task remains intriguing and difficult to explain. Future studies will be needed to understand these results.

As previously introduced, PA-TMS recruits neural structures from the targeted M1, and descending volleys travel through the corticospinal tract (Siebner et al., 2022). Differently, AP-TMS would recruit other neural circuits in M1 (Aberra et al., 2020) including corticocortical interneurons originating from the premotor cortex/SMA (Di Lazzaro and Rothwell, 2014). In modeling study, AP-TMS leads to an anterior spatial shift of the site of neural activation compared with PA-TMS in the direction of the premotor areas when the hand M1 area is targeted (Aberra et al., 2020). Considering that we targeted the LES M1 representation which is more medial than the hand M1 representation (Penfield and Boldrey, 1937; Roux et al., 2018), it is likely that the AP-TMS electric current also interact with neurons/interneurons from the SMA. Indeed, the SMA lies on the medial surface of the hemisphere (Tanji, 1994) sharing connections with the medial and rostral M1 (Picard and Strick, 1996, 2001, Nachev et al., 2008) embedding the M1 representation of the trunk (Penfield and Boldrey, 1937). Although this is an indirect observation, electrical stimulation of the premotor cortex in macaques evokes I-wave responses smaller and later than those evoked by M1 stimulation similarly as the later I-wave evokes by AP-TMS volleys (Shimazu, 2004). SMA/premotor areas can indirectly influence corticospinal motor output via dense corticocortical connections to M1 (Nachev et al., 2008), but also directly via descending, di-synaptic projection to spinal motoneurons (Dum, 2005). Thus, it is possible that when targeting M1-trunk representation, AP-TMS recruit neural elements from the premotor/SMA regions. Although speculative, this hypothesis could contribute to explain our results.

### Diverse neural circuits contribute to the activation of LES in the postural task

Multiple evidence support that premotor areas and the SMA contribute to anticipatory postural adjustment control (Massion, 1992; Viallet et al., 1992). For example, anticipatory postural adjustments are altered when: (1) a brain lesion affects the SMA (Massion et al., 1989), (2) a virtual lesion impacts SMA excitability using repetitive TMS in humans (Jacobs et al., 2009), and (3) GABA_A_ agonist is injected in SMA in monkeys (Takakusaki, 2017). Furthermore, a study by Yani et al. (2019) found that high-frequency repetitive TMS over the SMA decreased pelvic floor muscle tone and increased SMA activity, while low-frequency repetitive TMS had the opposite effects, suggesting that the SMA may play a role in regulating the inhibition–excitation balance in motor control. Thus, it is possible that the change in AP-MEP amplitude observed during postural task reflects the recruitment of circuits belonging to the premotor cortex/SMA (Aberra et al., 2020). This result adds to the similar increase of PA-MEP amplitude already observed by others (Chiou et al., 2016, 2018a; Massé-Alarie et al., 2018; Rowland et al., 2021) suggesting that circuits recruited by AP- and PA-TMS (allegedly from SMA/premotor and M1, respectively) could both contribute to control of the postural task. Also, although there was no difference in SICI between current directions, PA-SICI was significantly reduced compared with baseline, which is in line with Chiou et al. (2018a), suggesting a cortical contribution to postural control of back muscles. Subcortical structures (e.g., medullary reticular formation) projecting to the motoneurons through the extrapyramidal tract (e.g., reticulospinal) have been suggested to be also involved in postural control (Deliagina et al., 2008; Galea et al., 2010) and to receive and process motor information from the cerebellum, basal ganglia, and cortical areas (Takakusaki, 2017). The increase in R1 amplitude during the execution period could partially represent the excitatory influence of pyramidal and/or extrapyramidal pathways even though future studies will need to test this hypothesis. The cortical contribution observed in the current study could share similar mechanisms as those underlying crossed facilitation phenomenon of back muscles. Indeed, this phenomenon refers to the increase of the excitability of the corticospinal projection to a given muscle while another muscle is contracted rather than at rest (Perez and Cohen, 2008). For example, the contraction of an arm muscle increased the MEP amplitude of thoracic back muscles maintained at rest, and these mechanisms have been shown to be mediated, in part, cortically (Davey et al., 2002, Chiou et al., 2018b, 2020). Altogether, our results suggest that various neural circuits may contribute to the control of LES during a postural task.

### M1 and the corticospinal tract potential contribution in the voluntary control of LES

M1 and the corticospinal tract are described as fundamental structures of voluntary control of limb muscles in humans (Massion, 1992; Lemon, 2008). This hypothesis is frequently extrapolated to axial muscles (Ferbert et al., 1992; Jean-Charles et al., 2017) despite smaller cortical motor representation (Penfield and Boldrey, 1937; Boendermaker et al., 2014). However, the results of the current study suggest a minimal involvement of M1 and of the corticospinal tract in the voluntary control of LES. This might be explained by differences in motor descending pathway organization and function between distal limb and trunk muscles. Lawrence and Kuypers (1968a,b) performed lesions of the pyramidal (Lawrence and Kuypers, 1968a) and extrapyramidal tracts (Lawrence and Kuypers, 1968b) in rhesus monkeys and observed distinct motor impairments. A lesion of the pyramidal tract reduced the ability of the monkeys to use their fingers without postural impairment (Lawrence and Kuypers, 1968a). In contrast, the lesion of the ventromedial or lateral brainstem descending pathways resulted in an inability to right, a deficit in proximal movement, and when sitting the monkeys tended to slump forward (Lawrence and Kuypers, 1968b) suggesting, inter alia, an alteration in the control and tone of low back extensors. The voluntary task used in this study requires participants to “righten” by activating the low back extensors. Thus, it is important to consider that M1 likely contribute to voluntary control of back muscles as TMS over M1 elicits MEPs (Nowicky et al., 2001; Cariga et al., 2002; Davey et al., 2004; Chiou et al., 2016). However, considering that a minimal increase in MEP amplitude was observed in the execution of the voluntary task, our voluntary task may be predominantly controlled by extrapyramidal (e.g., ventromedian system) pathways with limited contribution of M1 and the corticospinal tract. In line with our results, postural (i.e., bilateral flexion of shoulders) or automatic (i.e., forced expiration during breath holding) activation of erector spinae muscles led to larger increase in MEP amplitudes compared with voluntary extension of the back in different studies (Nowicky et al., 2001; Chiou et al., 2016). Although these results need to be interpreted with caution due to different experimental contexts, they align with our current results.

An increase in MEP amplitude may be driven by an increase in M1 and/or motoneuronal excitability (Thompson et al., 1991; Mazzocchio et al., 1994; Ugawa et al., 1995; Davey et al., 1996). We observed an increase in R1 amplitude prior to the EMG onset of LES during the voluntary task and a minimal increase in MEP amplitude. Similarly, the H-reflex amplitude also increases prior to a voluntary contraction of the soleus (Petersen et al., 2009). The increase in motoneuronal excitability without change in MEP amplitude might reflect, at least partly, an increase of the spinal motoneurons excitability by descending pathways (Pierrot-Deseilligny et al., 1971; Crone and Nielsen, 1989; Nielsen and Kagamihara, 1993; Petersen et al., 2009), most likely extrapyramidal. A limited contribution of the corticospinal tract during voluntary task seems to be supported by recent TMS studies of back muscles. The motor learning of a complex visuomotor pelvic tilt task did not influence TMS outcomes suggesting that the neural circuits underlying motor learning do not lie within M1 representation of back muscles (Cavaleri et al., 2020; Shraim et al., 2022). For example, changes in nontested neural structures such as propriospinal and interneuronal circuits (Pierrotdeseilligny, 1996), extrapyramidal pathways—reticulospinal (Deliagina et al., 2008; Galea et al., 2010)—and premotor/SMA (Takakusaki, 2017) could be possible.

### Limitations

TMS of trunk muscles remains challenging because of the small cortical representations necessitating the use of a less focal coil (double-cone coil); the magnetic field may interact with more distant neural circuits in comparison with a figure-of-eight coil (Deng et al., 2013). However, considering similar results were obtained using a figure-of-eight and a double-cone coils (e.g., motor threshold; Desmons et al., 2021; Shraim et al., 2022), it is unlikely that the use of a double-cone coil significantly biased our results. Moreover, it was necessary to maintain a slight contraction of LES. Active contraction in addition to the task performed during neurophysiological testing complexifies the interpretation. Stretch reflex technique presents some shortcomings such as potential variability between taps due to experimenter and participant movement, and the test–retest reliability of this technique is not known. However, this is one of the only techniques that may test excitability of motoneurons projecting to back muscles which is critical to better understand the origin of the change in MEP size. The mere reversal of the coil to produce different current directions underestimates the complexity of M1. Identifying the exact stimulation location on individual participants remains challenging due to variations in cortical anatomy and template adaptation limitations. A more selective approach of the M1 nonuniform orientation may be more appropriate even though more technically challenging. The variability in MEP amplitude needs to be considered. Indeed, AP-MEP amplitudes are more variable than PA-MEP amplitudes (Hamada et al., 2013). To limit the effect of variability on the results, we employed an average of 15 MEPs per condition, as it has been shown to improve reliability (Cavaleri et al., 2017b). Finally, fatigue was not objectively measured. However, since the order of stimulation was randomized and participants were allowed to rest whenever they wished with no time limit, the potential impact of fatigue on the results was minimized.

## Conclusion

The present study aimed to investigate the effect of current direction on the neural control of the erector spinae muscles during the preparation and execution of different motor tasks. During the execution, we observed a specific increase in MEP amplitude from preparation to execution only while using AP-TMS in the voluntary task, whereas MEP change was present using both current directions in the postural task. We propose that the neural circuits and descending pathways involved in the motor control of the postural and the voluntary tasks are different. Surprisingly, M1 and the corticospinal tract seem to contribute more to the control of the postural than voluntary task. Future studies are needed to further understand how the nervous system controls LES.

## Synthesis

Reviewing Editor: David Franklin, Technische Universitat Munchen Decisions are customarily a result of the Reviewing Editor and the peer reviewers coming together and discussing their recommendations until a consensus is reached. When revisions are invited, a fact-based synthesis statement explaining their decision and outlining what is needed to prepare a revision will be listed below. The following reviewer(s) agreed to reveal their identity: Paul Strutton.

Your manuscript has been reviewed by two experts in the field. They both have interest in the manuscript and think that this is an important study of the neural control of trunk muscles. As you will see, both reviewers were positive about the study, but raise a number of concerns and suggestions regarding the current version of the study. We all see significant issues with the level of data presentation and the details provided within the manuscript. Specifically, the limited number of human studies cited in the introduction on trunk control, the inclusion of raw EMG presented similarly to the MEP data (with specific concerns about background levels and changes in excitability levels), the variability in reaction times for single sweeps, location of hotspots, and the inclusion of some participants with 30% MVC. In addition, the reviewers raise additional comments on both the introduction and discussion that should be addressed. I encourage you to revise your manuscript after addressing each of the concerns raised by the reviewers.

General comments from the reviewers.

The complex nature of the neural control of the trunk muscles is not well understood. The trunk muscles have multiple functions (posture, voluntary control) and the work described in this paper adds to the body of literature on the neural control of the trunk muscles in different tasks. The manuscript uses single-pulse and paired-pulse TMS in a clever way to engage different cortical control circuits. It compares the induced responses across different phases (preparation and execution) of postural and voluntary tasks. These comparisons allowed the authors to suggest for the first time the dominant role of premotor/SMA, compared to M1, during the execution of voluntary tasks. This finding challenges the common knowledge that premotor/SMA is more involved in postural control during voluntary movements, and premotor/SMA provides a redundant circuit of voluntary control when pathology affecting M1 occurs. I think this research opens the door to more TMS and other neuroimaging research that addresses the roles of premotor, M1, and inhibition-excitation balance in motor control.

Reviewer 1:

This study set out to examine the neural control of the lumbar erector spinae (LES) muscles using transcranial magnetic stimulation over the motor cortex using current induced in the brain in posterior anterior (PA) and anteroposterior (AP) directions. Participants undertook 2 tasks involving activation of the trunk, one postural (shoulder flexion) and one voluntary (pelvic tilt) in response to a visual cue and compared the sizes of the motor evoked responses (MEPs). Stimulation (TMS, and taps to induce stretch reflexes) was delivered after a warning signal (250ms before a go signal) and after the go signal (30ms before the onset of electromyographic (EMG) activity) in the LES. Results showed that corticospinal excitability was higher in the postural task during the execution period (after the go signal, but before the onset of EMG in the LES) than the preparation period (after the warning signal, but before the go signal) irrespective of current direction. Further, in the voluntary task, MEP amplitude was higher with AP current direction during the execution period than the preparation period and than PA-MEP during execution period. Short-interval cortical inhibition (SICI) with PA current direction was lower in preparation than execution periods. The authors conclude that PA- and AP-TMS neural circuits are differently involved in the control of LES and suggest caution when recognising the importance of the primary motor cortex in the voluntary control of ES.

The study is generally well written and experiments diligently performed and data presented well clearly. The rationale for the study is sound; current direction has been suggested to activate different neuronal populations and this has been demonstrated in a static task for the trunk muscles, so it seems reasonable to study this in tasks involving the trunk which have differing degrees of postural involvement (shoulder flexion [more] vs pelvic tilt [less]). The work adds to the body of literature on the neural control of the trunk muscles in different tasks.

I have some comments on the content of the introduction, as there is an abundance of evidence on the control of the trunk muscle in humans. The discussion also frequently states that M1 and the corticospinal tract are minimally involved in the voluntary control of the trunk. I disagree with this sentiment.

Introduction

Lines 42-45. I am unclear as to why the introduction states that the knowledge framework on neural control of the trunk is often based on limb muscles. There is plenty of evidence from TMS studies on the trunk unrelated to limb muscles, much of this evidence is already cited in the paper, so I'm slightly confused by this statement.

I'm not sure why the experiment was divided into two parts and only 9 participants undertook both parts. The rationale for the study was to test responses in postural and voluntary tasks, why did participants not undertake the full experiment (i.e. both parts), this would seem entirely sensible, no?

It would be good to see data for raw (rectified) EMG presented in a similar fashion to the MEP data, so that it is possible to appreciate the levels of ongoing EMG prior to the stimulus presentation in each of the 2 tasks. Figure 6 gives an indication of this, but the trace starts at the warning signal, what is the EMG level prior to the warning signal, i.e. what is the effect of the warning signal on background EMG activity?

I think more details should be provided about the actual timings of the TMS pulses 30ms before the onset of the rise in EMG. It is stated that LES EMG onset was measured for each participant in two blocks of 15 TMS (5 in the preparation period, 5 in the execution period, 5 baseline). Presumably there were therefore 3 average reaction times calculated, one for each of the 3 periods in which it was tested? However, during the experiment the TMS would have been delivered at 30ms prior to each of these averages. I therefore assume that due to the variability in reaction times for single sweeps, some stimuli would have been delivered earlier than 30ms and some later than 30ms before the onset of rise in EMG activity? How was this addressed? Was there a bin width (e.g. 25-35ms before onset of EMG) into which TMS pulses were accepted as having been delivered at ~30ms prior to the rise in EMG, something similar to that performed previously (see data analysis in Chiou et al., 2018).

Were the hotspot locations for PA and AP stimulation different?

I would suggest modifying the y-axes labels for Fig 3a, 4a and 5, to MEP amplitude (% change from baseline) as this better reflects the measure and is consistent with what is stated in the figure legends.

The latencies of the R1 responses were stated as being 12-22ms (lines 245-246), but reflex latencies they have also been reported as being ~5.5ms in the ES muscles from taps applied to the muscle or the spinous process (Kuppuswmay et al., 2005). The latencies of the responses are not reported in the paper (only averages from which time window duration was determined for analysis); it would be good to see how the latencies of the reflex responses compare to that published.

Lines 352-354 - "For two participants, it was difficult to evoke consistent MEPs at 15% MVC, thus 30% MVC was used throughout the session." Why were these 2 participants included, as the facilitation of the corticospinal tract would increase between 15% MVC and 30% MVC so MEPs would not be comparable?

Discussion

The discussion frequently states that the corticospinal tract is minimally involved in the voluntary control of ES. Indeed, there is a section headed "M1 and the corticospinal tract are minimally involved in the voluntary control of LES". I would suggest that this is not the case.

Lines 535-536. I would suggest including some work on corticospinal control of erector spinae muscles here, by Nowicky et al., 2001, as this provides evidence of involvement of the corticospinal pathway in voluntary control of the ES. This is in contrast to the assertion that M1 is minimally involved in voluntary control of ES.

Line 558-550. "The limited contribution of the corticospinal tract during voluntary task is..." I would suggest that this is not the case, as there is plenty of evidence that the corticospinal tract is involved in voluntary control of the back muscles (Cariga et al., 2004; Chiou et al., 2016; Davey et al., 2004; Nowicky et al., 2001).

Conclusion

Line 589-591. I am perplexed at this last sentence, as it has already been shown that corticospinal control of ES is higher in a postural rather than a voluntary task (Chiou et al., 2016).

Minor comments

The formatting of the references in the reference list needs checking, there are some inconsistencies (e.g. full names for Aruin, but not for others, some references in bold, others not, some have page numbers, others don't).

Abstract

Line 12 - "pelvic" should be "pelvis"

Lines 20-21 - I do not follow the assertion that "PA-TMS circuit seems mostly involved in the postural task" given the result that during the postural task MEPs were larger (in execution than preparation) irrespective of current direction and that "AP-TMS circuit seems involved in both tasks" when AP-MEPs were larger than PA-MEPs in the voluntary task.

Significance statement

Lines 28-29 - "allowed to determine if different neuronal circuits contribute to postural and voluntary control of back muscles". I would suggest rewriting this sentence. Maybe - "allowed examination of the contributions of different neuronal circuits to postural and voluntary control of back muscles"

Lines 34-35. "It suggests - against current knowledge - a smaller contribution of the corticospinal tract in voluntary than in postural control." I would say that there is enough evidence (already cited in the paper) that there is a smaller contribution of the corticospinal tract to voluntary rather than postural control of the ES muscles (see Chiou et al., 2016 and 2018).

Introduction

Line 38 - "presents" should be "present"

Materials and Methods

Line 186 - "A ground electrode (9160F, 3M, Saint Paul, 187 USA) was positioned over the right anterosuperior iliac spine and iliac crest" - Minor point, but these are 2 distinct anatomical locations, was one ground electrode placed in one location in some participants and in the other in other participants?

Line 212 - Add "coil" after "double cone".

Line 216 - Format of reference - "Nowicky A.V", should just be "Nowicky".

Line 302 - Change "Post hoc were" to "Post hoc tests were".

Results

Line 350 - Change "realised" to "undertook" or "performed".

Discussion

Line 482-282. "Differently, AP-TMS would recruit other neural circuits in M1 (Aberra et al., 2020) whose cortico-cortical interneurons from the premotor cortex/SMA (Di Lazzaro and Rothwell, 2014)." This sentence appears to be missing a word.

Line 490 - Incorrect spelling - "connexion".

Line 531 - "structure" should be "structures"

Line 540 - "tract" should be "tracts"

References:

Cariga P, Catley M, Nowicky AV, Savic G, Ellaway PH, Davey NJ. (2002). Segmental recording of cortical motor evoked potentials from thoracic paravertebral myotomes in complete spinal cord injury. Spine (Phila Pa 1976). 2002 Jul 1;27(13):1438-43. doi: 10.1097/00007632-200207010-00013.

Chiou S-Y, Gottardi SEA, Hodges PW, Strutton PH (2016) Corticospinal Excitability of Trunk Muscles during Different Postural Tasks. PLoS ONE 11:e0147650.

Chiou S-Y, Hurry M, Reed T, Quek JX, Strutton PH (2018) Cortical contributions to anticipatory postural adjustments in the trunk: Cortical involvement in postural adjustments. J Physiol 596:1295-1306.

Davey NJ, Rawlinson SR, Nowicky AV, McGregor AH, Dubois K, Strutton PH, Schroter RC. (2004). Human corticospinal excitability in microgravity and hypergravity during parabolic flight. Aviat Space Environ Med. 2004 Apr;75(4):359-63.

Kuppuswamy A, Theodorou S, Catley M, Strutton PH, Ellaway PH, McGregor AH, Davey, NJ (2005). Motor neurone excitability in back muscles assessed using mechanically evoked reflexes in spinal cord injured patients. J Neurol Neurosurg Psychiat; 76(9):1259-63. doi: 10.1136/jnnp.2004.045021.

Nowicky AV, McGregor AH, Davey NJ. (2001). Corticospinal control of human erector spinae muscles. Motor Control. 5(3):270-80. doi: 10.1123/mcj.5.3.270.

Reviewer 2:

This research focuses on corticomotor control of lumbar erector spinae in postural and voluntary tasks. It uses single-pulse and paired-pulse TMS in a clever way to engage different cortical control circuits. It compares the induced responses across different phases (preparation and execution) of postural and voluntary tasks. These comparisons allowed the authors to suggest for the first time the dominant role of premotor/SMA, compared to M1, during the execution of voluntary tasks.

This work is complex as it had to consider many competing factors. I truly appreciate it; however, my main concern is the clarity of the study design. I included my comments section by section as they were presented in the combined file I reviewed.

Figure 1: Please review the legend to be clear. I could not understand the experimental setup, which is key. For example:

> It is hard to tell exactly what the tasks were, and the legend does not describe them. Please describe the tasks instead of only using the terms "postural" and "voluntary"

> It was clear how you provided stimuli 250 ms prior to the Go signal since it was preset to be 1500 ms after the warning signal; however, I do not understand how you could determine 30 ms prior to the onset of EMG to stimulate (hence the "Execution" condition).

> Since you have repeated stimulations, the figure and the legend should explain the inter-stimulus interval (ISI). If this is not the case and you have repeated trials, please indicate that.

> Also, the double cone coil and coordinates of the stimulation sit(e) should be listed.

Figure 2:

> How many traces are there in each plot?

> Is R1 (short latency response) the same as SR (stretch reflex)?

> It seems that the listed stimulation paradigms were single-pulse TMS (PA-TMS, AP-TMS). Where are the responses to the "3 series of 15 paired pulses" listed under TMS in Figure 1-D?

Figure 3:

> % of change relative to the Baseline may not work if you cannot induce responses during the Baseline condition. For example, in Figure 2, Baseline/Postural/R1, I cannot tell if there was a response in the presented data. Please explain whether this situation happened and how you calculated the % change in that event. The same issue applies to the results presented in Figure 4.

> In the legend, please explain which differences were statistically significant.

> The vertical "preparation" and "execution" do not align with the labels and ticks on the x access of Figure 3.a. Also, I do not understand why you would compare two different conditions using two different TMS current directions. What is the purpose of that comparison? Are you referring to an interaction term? if yes, please indicate that in the figure legend.

> Instead of using the standard deviation for the error bars, I suggest using the 95% confidence interval to determine whether a difference is significant. The same applies to Figure 4.

Figure 4:

> In the legend, please explain which differences were significant.

Figure 5:

> In the legend, please explain which differences were statistically significant.

> Subsetting was not justified/explained in the figure legend.

Figures 3-5:

> There is no clear message/title explaining the key results.

Figure 6:

> I appreciate the information in this figure; however, can you clarify the following:

>> Whether this is a group or individual data

>> Whether each signal is made of a continuous EMG signal or concatenated EMG short signals.

>> Whether these EMG responses are the average of AP and PA responses.

Abstract

There are issues with the abstract. These issues include the lack of clarity regarding the research question, study design, results, and conclusions.

> Lines 2-6: While you are specific about the effect PA-TMS, what did you mean by the term "probe" when discussing the effect of AP-TMS.

> Lines 6-7: "Neural circuits activated by....." is unclear. I believe neural circuits in motor control listed above are already active. TMS may just enhance or prohibit their effects. While we apply both TMS (PA and AP) to M1, PA-TMS may cause an excitatory effect via depolarization of the pyramidal neurons, AP-TMS may cause some kind of an effect (possibly inhibitory) by targeting the already active connection from the premotor cortex to the primary motor cortex.

> Lines 8-9: The use of "PA- and AP-TMS neural circuits" is wrong. TMS does not have circuits. TMS interacts with already active networks, and the associated effect observed at the EMG level is net without directly indicating which circuits at the brain level were involved.

> Lines 10-14: Please explain the site(s) of the paired-pulse TMS.

> Lines 10-14: I do not understand the connection between AP-TMS or PA-TMS and paired-pulse TMS. Please explain, especially given that a double cone coil is used in two opposite directions, as shown in Figure 1.

> Lines 10-14: I can understand when baseline and preparation TMS were applied; however, I do not understand how you could figure out when to apply TMS before the onset of the LES activation (execution period). Please clarify.

> Lines 15-19: Please describe the measurements before discussing the results.

> Lines 15-19: Using "modulation" in TMS can mean many things. Please define modulation after you describe the measurements of interest.

> Lines 15-19: You compared corticospinal excitability modulation and MEP amplitude. It is confusing to the reader to use "corticospinal excitability" and "amplitude" because they are sometimes understood as equal.

> Line 16: Is the p-value (p=0.0002) the same for the two comparisons implied?

> Lines 20-23: This paragraph is vague and not clear. Please revise and support with evidence.

> Lines 27-29: You should be very cautious with your conclusions. For example, AP-TMS and PA-TMS may have differential effects while engaging the same circuit(s) under the same control condition. Thus, you may be unable to provide evidence of an association between a specific circuit and a specific control condition.

> Lines 30-34: You should be specific because you measured corticospinal excitability during preparation and execution.

> Lines 30-33: The results did not compare changes/modulation in corticospinal excitability, measured with PA-TMS, during the preparation and execution phases of the voluntary task. Please add this result to the results section.

> Lines 33-35: This is a result. You should make it clear and report it in the results section. I also do not understand the associated conclusion since the result is unclear.

Introduction

I do not think you provide a clear argument why AP-TMS can be used to test the contribution of premotor areas in the control of LES. Also, why do you focus on one muscle while a movement usually includes a synergy of muscles? Furthermore, the use of the term "modulation" is troublesome. Modulation implies lasting changes (plasticity) which is not the case in this study. Finally, you do not mention the reliability of AP-TMS and PA-TMS. TMS responses are known to be highly variable, even within the same person and the same protocol (you can check work by Hamada et al. and others).

> Lines 49-50, 55-56: Please explain the results from the 1992 and 2007 studies. I believe these are old studies and the only of human subjects. Thus, referencing them requires more details.

> Line 63: Please do not assume that the reader understands SICI. Describe SICI (short-latency intracortical inhibition) and explain the possible role of AP-TMS in engaging certain interneurons.

Also, what is the difference between profound SICI and higher motor thresholds? I think a profound SICI and higher motor threshold are similar or at least indistinguishable when looking at the characteristics of the MEPs.

> Line 67: These cited TMS studies do not provide evidence to support this conclusion. TMS is not very specific. Double-cone coils are stronger than figure-of-8 coils that are used in hand studies. Double-cone coils may engage the two circuits you are referring to regardless of the direction of the TMS coil/current.

> Lines 76-78: What do you mean by this sentence?

> Lines 81-84: I do not think you provide a clear argument why AP-TMS can be used to test the contribution of premotor areas in the control of LES.

> Line 90: You talk about intra-cortical excitability, yet in the above paragraphs, you mentioned intra-cortical inhibition. Edit the introduction to be consistent unless I am missing your hypothesis.

> Lines 92-95: I think the term "modulation" in your hypotheses is misleading. Single-pulse does not cause modulation; however, changing the current direction may result in changes in the characteristics of the MEPs. These changes should not be called modulation since modulation implies lasting changes (plasticity).

Methods

This section is the core of this study, and its complexity maked it hard to read. As it is now, it requires reorganization and clarification.

Experimental study design:

> Both movements/tasks include different muscles, including LES. Motor control strategies may change across participants. Trunk/back muscles, including the LES, can have overlapping representations in the medial motor cortex (for example, Yani et al., 2018 in Scientific Reports showed overlapping representations of pelvic floor muscles (postural) and gluteal muscles (prime mover) in both SMA and M1). A double-cone coil may not differentiate between these presentations. Why did you not consider other muscles when addressing your research question regarding the contribution of premotor areas to the control of LES?

Study design for both experiments:

> Why did you start with the maintained pre-activation of LES? Was that because you needed to create baseline responses for normalization? If so, the % MVC during baseline, preparation, and execution should be accounted for in the analyses.

> Line 150: What are the TMS outcomes?

> Line 153: I do not understand how you were able to achieve the second condition ("during the execution period, -30 ms prior to the LES EMG onset) without preassuming the time onset of the EMG. Please explain. The method explained (Lines 160-171) was not clear, and the choice of -30 ms was not justified.

> Lines 172-180: Can you provide the randomization sequences and justify its need in this small sample?

> Lines 210-227: Can you provide information about the pre-activation intensity used while deciding the AMT? Also, please provide a table with the MNI coordinates. Other researchers can use it in the future instead of hot-spotting.

> Lines 228-232: Please explicitly list the location of the conditioning stimulus (I assume the same hot spot in M1).

> Lines 234-251: I cannot comment on this part.

Data analysis:

> Lines 271-287: I cannot comment on this part.

> Lines 288-293: I do not understand this section. Why was one 50-ms epoch chosen? What were the purpose and the analysis?

Statistical analysis:

> Lines 296-340: I am unfamiliar with nparLD (nparLD package), and I cannot comment on this part.

Results

The clarity in reporting the results is needed. When describing the results, please list the corresponding table(s), not just the figures.

Table 1:

> This ANOVA table is hard to read.

> Change the width of the columns and possibly edit the "Figure" column. I am unsure if "Table 2" listed under this column is correct.

> Also, place the RTE results in a location that is not confusing.

> Defined within the table all variables (Normalised MEP, Normalised SICI, Normalised R1 amplitude).

Table 2:

> This table should become Table 1 based on the reporting of the results.

> Define with the table "Reaction time (ms)", "PA-AMT (% MSO)", and "AP-AMT (% MSO)".

Table 3 and Table 4:

> Defined within the table all variables.

> Try to stay away from the term modulation. I would use "changes" in the collected variables.

> Lines 358-366: it is hard to believe that the latency is the same regardless of the period. Physiological variability should result in minimal differences, even when stimulating the same cortical hotspot and corticospinal tracks.

> Lines 374-377, 397-405, 421-428: It was unclear which values were due to AP-TMS and which were due to PA-TMS. When describing the results, please list the corresponding table(s), not just the figures.

> Lines 432-436: I do not understand the purpose of figure 6. How did you reach these results and use them in the main analyses?

Discussion

To support your claim that "corticospinal tract are minimally involved in the voluntary control of LES" please include studies with other neuroimaging modalities that shows higher and differential activation when comparing premotor/SMA to M1, for example, PET/task-fMRI/EEG. You make a point. I am a believer in the ability of premotor/SMA to control muscles directly; however, classic basic science research and pathology implicated premotor/SMA in postural control during voluntary movements and as redundant circuits of voluntary control in pathologic cases. Your conclusion (lines 585-587) supports that premotor/SMA circuits are active during the postural control task (changes in MEP using AP-TMS) and voluntary task (changes in MEP using AP-TMS).

> Lines 449-455: I am not sure if I agree with your conclusion of a smaller contribution of the corticospinal tract in the voluntary task compared to the postural task. Furthermore, the "execution" condition occurred 30 ms before the onset of the EMG, which is a long period for engaging the corticospinal tract. I am unsure of the relevance of observing a higher peak EMG when comparing the voluntary task to the postural task.

> Lines 464-466: I understand that SMA and Premotor may have direct corticospinal projections to control LES. However, you only stimulated M1 while using single and paired-pulse TMS. Thus, it is hard to differentiate the contribution of SMA/Premotor from the contribution of M1, since M1 receives projections from SMA and Premotor and is considered the main gateway of motor outcome in voluntary control. Furthermore, you based this conclusion on only the assumption derived from two studies (Aberra et al., 2020; Di Lazzaro and Rothwell, 2014). I think further explanation is needed.

> Line 471-479: You finally explain the excitation of inhibitory circuits (interneurons). You should bring this up early in the manuscript to make it clear to the reader. TMS is state-dependent and engages active circuits; thus, observing PA-SICI during both tasks and observing no AP-SICI during both tasks may suggest that PA-TMS should also involve premotor/SMA circuits and that these circuits are involved in both tasks. This supports the comment I made above regarding your conclusion of the differential contribution of SMA/Premotor and M1 (Lines 464-466).

> Lines 480-497: This paragraph's end discusses this study's limitation, which is only stimulating M1. Whether the signal (current) spread to premotor/SMA or AP-TMS protocol activated the projections from premotor/SMA to M1 is impossible to argue in this study.

> Lines 530-566: To support your claim that "corticospinal tract are minimally involved in the voluntary control of LES" please include studies with other neuroimaging modalities that shows higher and differential activation when comparing premotor/SMA to M1, for example, PET/task-fMRI/EEG (for example Yani et al, 2019 in Neurourology and Urodynamics used 2 opposing rTMS applied to SMA and showed that HF-rTMS reduced muscle tone and increased SMA rs-fMRI activity, while LF-rTMS caused opposite effects, suggesting the possible role of inhibition-excitation balance in motor control). You make a good point. I am a believer in the ability of premotor/SMA to control muscles directly; however, classic basic science research and pathology implicated premotor/SMA in postural control during voluntary movements and as redundant circuits of voluntary control in pathologic cases. Your conclusion (lines 585-587) supports that premotor/SMA circuits are active during the postural control task (changes in MEP using AP-TMS) and voluntary task (changes in MEP using AP-TMS).

> Lines 568-581: Please discuss the potential limitation caused by variability in TMS responses. It would be appreciated if you could provide individual data supporting that all participants showed similar trends in the responses to AP-TMS, PA-TMS, R1.
